# The Ribosome Biogenesis Protein Nol9 Is Essential for Definitive Hematopoiesis and Pancreas Morphogenesis in Zebrafish

**DOI:** 10.1371/journal.pgen.1005677

**Published:** 2015-12-01

**Authors:** Ewa Bielczyk-Maczyńska, Laure Lam Hung, Lauren Ferreira, Tobias Fleischmann, Félix Weis, Antonio Fernández-Pevida, Steven A. Harvey, Neha Wali, Alan J. Warren, Inês Barroso, Derek L. Stemple, Ana Cvejic

**Affiliations:** 1 Department of Haematology, University of Cambridge, Cambridge, United Kingdom; 2 Wellcome Trust Sanger Institute, Genome Campus, Hinxton, Cambridge, United Kingdom; 3 NHS Blood and Transplant, Cambridge, United Kingdom; 4 Cambridge Institute for Medical Research, Cambridge, United Kingdom; 5 Wellcome Trust-Medical Research Council Stem Cell Institute, University of Cambridge, Cambridge, United Kingdom; Yale Univ. School of Medicine, UNITED STATES

## Abstract

Ribosome biogenesis is a ubiquitous and essential process in cells. Defects in ribosome biogenesis and function result in a group of human disorders, collectively known as ribosomopathies. In this study, we describe a zebrafish mutant with a loss-of-function mutation in *nol9*, a gene that encodes a non-ribosomal protein involved in rRNA processing. *nol9* ^*sa1022/sa1022*^ mutants have a defect in 28S rRNA processing. The *nol9* ^*sa1022/sa1022*^ larvae display hypoplastic pancreas, liver and intestine and have decreased numbers of hematopoietic stem and progenitor cells (HSPCs), as well as definitive erythrocytes and lymphocytes. In addition, ultrastructural analysis revealed signs of pathological processes occurring in endothelial cells of the caudal vein, emphasizing the complexity of the phenotype observed in *nol9* ^*sa1022/sa1022*^ larvae. We further show that both the pancreatic and hematopoietic deficiencies in *nol9* ^*sa1022/sa1022*^ embryos were due to impaired cell proliferation of respective progenitor cells. Interestingly, genetic loss of Tp53 rescued the HSPCs but not the pancreatic defects. In contrast, activation of mRNA translation via the mTOR pathway by L-Leucine treatment did not revert the erythroid or pancreatic defects. Together, we present the *nol9* ^*sa1022/sa1022*^ mutant, a novel zebrafish ribosomopathy model, which recapitulates key human disease characteristics. The use of this genetically tractable model will enhance our understanding of the tissue-specific mechanisms following impaired ribosome biogenesis in the context of an intact vertebrate.

## Author Summary

The production of ribosomes, the protein-synthesizing machines, is fundamental in all cells. It is a very complex process that requires the coordinated actions of ribosomal and non-ribosomal proteins. Impairment of ribosome formation and function leads to a class of disorders known as “ribosomopathies”. Here, we describe the identification and characterization of a zebrafish mutant in *nol9*, a gene encoding a non-ribosomal protein involved in ribosome biogenesis. The *nol9* ^*sa1022/sa1022*^ mutants show defects in the exocrine pancreas and erythrocytes due to impaired cell proliferation. These phenotypic features of *nol9* ^*sa1022/sa1022*^ mutants are reminiscent of the clinical symptoms of Shwachman-Diamond syndrome, a ribosomopathy characterized by exocrine pancreatic insufficiency and hematopoietic defects. Interestingly, we found that hematopoiesis but not pancreas morphogenesis in *nol9* ^*sa1022/sa1022*^ larvae is Tp53-dependent, highlighting that the consequences of impaired ribosome biogenesis differ between tissues within the same organism. This study provides novel insight into the function of the ribosome biogenesis protein Nol9 in zebrafish development and presents a novel model that will help to decipher the tissue-specific mechanisms of ribosomopathies.

## Introduction

Ribosome biogenesis is a highly conserved and remarkably complex process that is essential for cell growth and proliferation. It utilizes 60% of total cellular transcription in a growing yeast cell, with 2,000 ribosomes synthesized every minute [1]. It requires the coordinated action of all three RNA polymerases (RNAP I, II, III) and the synthesis of 4 ribosomal RNAs (rRNAs), 82 core ribosomal proteins (RPs), more than 200 non-ribosomal proteins and approximately 70 small nucleolar RNAs (snoRNAs) [2]. While complete loss of expression of genes encoding ribosomal components has not been described in humans, haploinsufficiency or partial loss of protein expression of some ribosome biogenesis proteins has been shown to be a common basis of the group of disorders known collectively as “ribosomopathies” [3]. Although all ribosomopathies involve ribosomal dysfunction, they show different modes of inheritance and the clinical presentations may vary even among patients carrying the same mutation. Clinical features of the ribosomopathies include defects in growth and development, craniofacial and skeletal defects, hematological abnormalities with increased propensity to the development of malignancies such as acute myeloid leukemia (AML) and myelodysplastic syndrome (MDS) [3]. Ribosomopathies that present with hematological defects include Diamond Blackfan anemia (DBA), 5q^-^ syndrome, Shwachman-Diamond syndrome (SDS) and T-cell acute lymphoblastic leukemia (T-ALL). Additional examples of ribosomopathies include Treacher Collins syndrome (TCS), isolated congenital asplenia (ICAS), aplasia cutis congenita (ACC), Bowen-Conradi syndrome (BCS), North American Indian Childhood cirrhosis (NAIC) and alopecia, neurological defects and endocrinopathy (ANE) syndrome [4].

One of the most intriguing aspects of ribosomopathies is that, despite the fact that they all share a common defect in the same biological process there is a high degree of cell and tissue-specific pathology [5]. Evidence from a number of model systems suggested activation of Tp53 [6–16] and mTOR [17, 18] pathways following ribosome dysfunction. However, modulation of these pathways had variable success in reverting tissue specific phenotypes in different models of ribosomopathies, again emphasizing the complexity of these disorders. This lack of basic insight into the molecular and biochemical defects that underlie ribosomopathies is reflected by a lack of effective therapeutic strategies, often resulting in poor outcomes for patients suffering from these disorders [3]. Current therapies include steroids and chronic transfusions for DBA patients and pancreatic enzyme replacement, granulocyte colony-stimulating factor, antibiotics and transfusion support for SDS patients. The only definitive treatment for the hematopoietic defects in DBA and SDS is bone marrow transplantation [19, 20]. However, graft failure, graft versus host disease and infection contribute to the substantial morbidity and mortality associated with these treatments [19, 21]. Therefore, there is a need to develop novel, targeted, therapeutic strategies based on understanding the pathophysiology of these disorders; which is why ongoing work to generate optimal *in vivo* models is essential for further progress in the field.

Here we report the identification and analysis of a zebrafish mutant with a loss-of-function mutation in the *nol9* gene. The human NOL9 protein is a polynucleotide 5’-kinase involved in ribosome biogenesis [22]. It is a non-ribosomal protein that is required for cleavage of the ITS2 region from a pre-rRNA to generate 5.8S and 28S rRNAs and for the synthesis of the 60S ribosomal subunit [22]. Similarly, the NOL9 homolog in *S*. *pombe*, Grc3, is required for pre-rRNA processing, particularly ITS2 processing [23]. In this study, we demonstrate that *nol9* ^*sa1022/sa1022*^ mutants have a defect in pre-rRNA processing within ITS2. As in the majority of congenital syndromes in human, zebrafish *nol9* ^*sa1022/sa1022*^ larvae had multiple organ failures including intestine, liver, pancreas and impaired definitive erythropoiesis and lymphopoiesis. These phenotypic features of *nol9* ^*sa1022/sa1022*^ mutants are reminiscent of the clinical symptoms of Shwachman-Diamond syndrome, an autosomal recessive ribosomopathy [24–27] that is characterized by exocrine pancreatic insufficiency, hematological abnormalities including neutropenia, anemia and thrombocytopenia and skeletal defects. Our detailed characterization of *nol9* ^*sa1022/sa1022*^ larvae further shows that both pancreatic and erythroid defects are due to impaired proliferation as well as differentiation of respective progenitor cell types. Additionally, we show that impaired erythropoiesis, but not pancreatic development, is rescued in double knock out *nol9* ^*sa1022/ sa1022*^ */tp53* ^*zdf1/zdf1*^ larvae.

## Results

### *nol9* ^*sa1022/sa1022*^ zebrafish mutants display impaired development of the intestine, liver, pancreas and definitive erythrocytes

**Fig 1:**
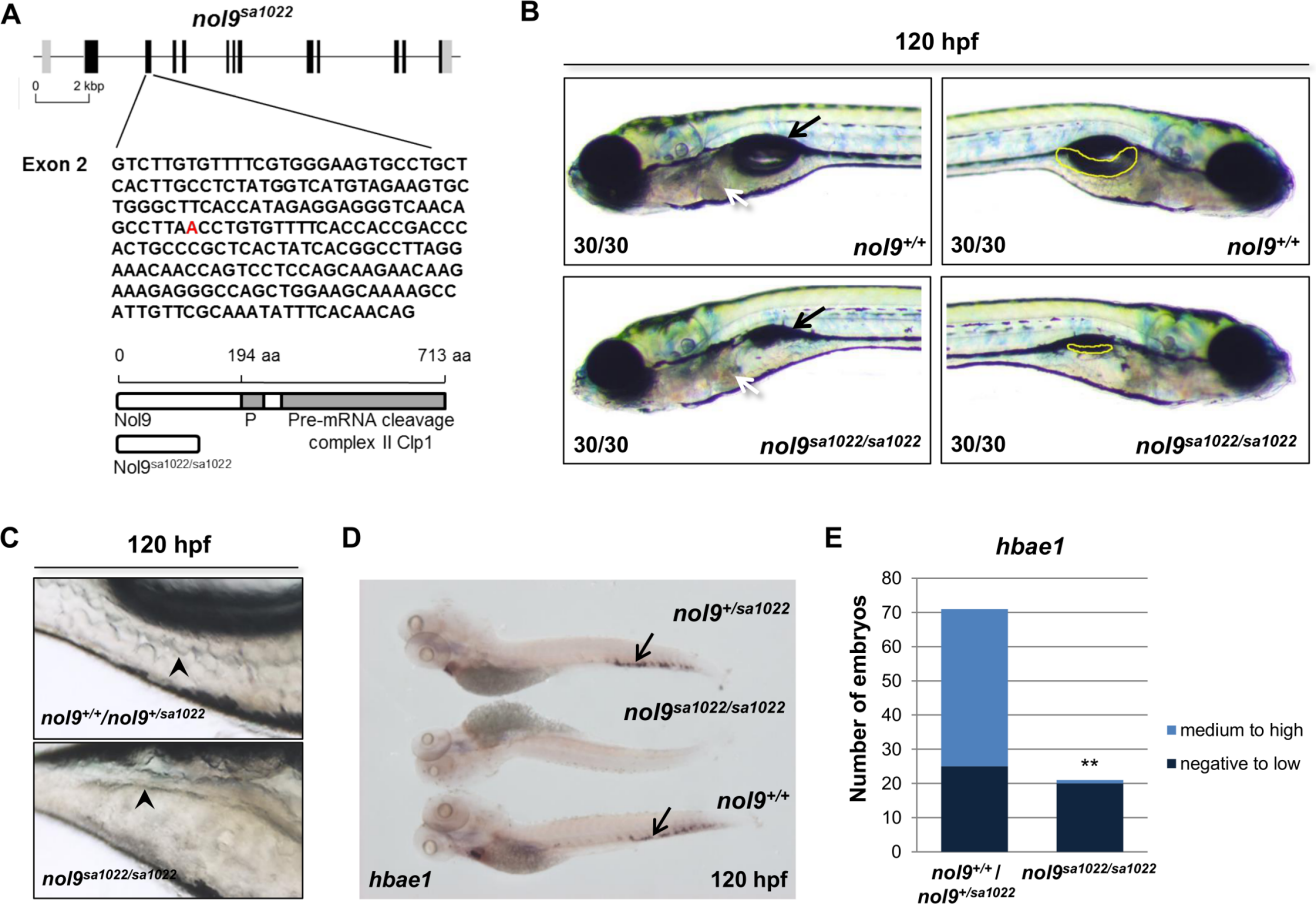
Loss-of-function *nol9* mutation leads to defects in the development of the intestine, liver, pancreas and definitive erythrocytes in zebrafish. (A) Schematic representation of the *nol9* gene and protein. Vertical bars represent exons (black boxes) and UTRs (grey boxes). The nonsense point mutation in *nol9* ^*sa1022*^ is marked in red. Wild-type Nol9 protein and its expected truncated version resulting from the *nol9* ^*sa1022*^ allele are shown. Domains of the Nol9 protein are indicated by grey boxes. P—P-loop NTP-ase domain. (B) Representative DIC images of *nol9* ^*+/+*^ and *nol9* ^*sa1022/sa1022*^ larvae at 120 hpf. Left and right sides of each larva are shown. White arrow points to the liver. Black arrow shows the swim bladder. Pancreas is outlined in yellow. (C) Representative bright filed images of the intestinal folds at 120 hpf. The *nol9* ^*sa1022/sa1022*^ mutant larvae have less developed intestinal folds (black arrowhead) compared to *nol9* ^*+/+*^ siblings. (D) Whole-mount *in situ* hybridization using *hbae1* riboprobe at 120 hpf. Arrow indicates *hbae1*-positive definitive erythrocytes present in the CHT of *nol9* ^*+/sa1022*^ and *nol9* ^*+/+*^ but not *nol9* ^*sa1022/sa1022*^ embryos. (E) Quantification of *hbae1 in situ* hybridization data. Data are represented as the number of larvae belonging to each phenotypic group. Fisher’s exact test, **, p<0.01.

The *nol9* ^*sa1022/sa1022*^ mutant was first identified in the Zebrafish Mutation Project, and it carries a nonsense mutation in codon 195 of the gene (Fig 1A) [28, 29]. *nol9* ^*sa1022/sa1022*^ embryos developed normally during the first 72 hpf with no obvious morphological differences when compared to heterozygous (*nol9* ^*+/sa1022*^) or wild-type (wt) siblings. However, at 96- and 120 hpf, the *nol9* ^*sa1022/sa1022*^ larvae lacked intestinal folds, had a smaller liver and pancreas and exhibited impaired yolk absorption compared to wt siblings (Figs 1B and 1C and S1A–S1C). Additionally, over 90% of the *nol9* ^*sa1022/sa1022*^ fish failed to inflate their swim bladder. The digestive organ phenotype was completely penetrant and the *nol9* ^*sa1022/sa1022*^ larvae died by 10 days post fertilization (dpf).

Some common features of ribosomopathies include craniofacial and hematological defects [30]. At 120 hpf, the *nol9* ^*sa1022/sa1022*^ larvae displayed normal jaw and branchial arch structures indicating that *nol9* is not required for normal skeletal development (S1D Fig). We also examined erythropoiesis in our model. Zebrafish erythropoiesis, similar to mammals, occurs in two sequential waves: primitive and definitive. While primitive erythropoiesis was intact at all the time points examined (48-, 96- and 120 hpf) as assessed by *o*-Dianisidine staining (S2A–S2D Fig), *nol9* ^*sa1022/sa1022*^ larvae displayed a dramatic decrease in the number of *hbae1-*positive definitive erythrocytes in the caudal hematopoietic tissue (CHT) at 96- (S2E and S2F Fig) and 120 hpf (Fig 1D and 1E). These results strongly suggest that *nol9* is critical for the production of definitive but not primitive erythrocytes.

### *nol9* expression is consistent with digestive organ and hematological defects of *nol9* ^*sa1022/sa1022*^ mutants

**Fig 2:**
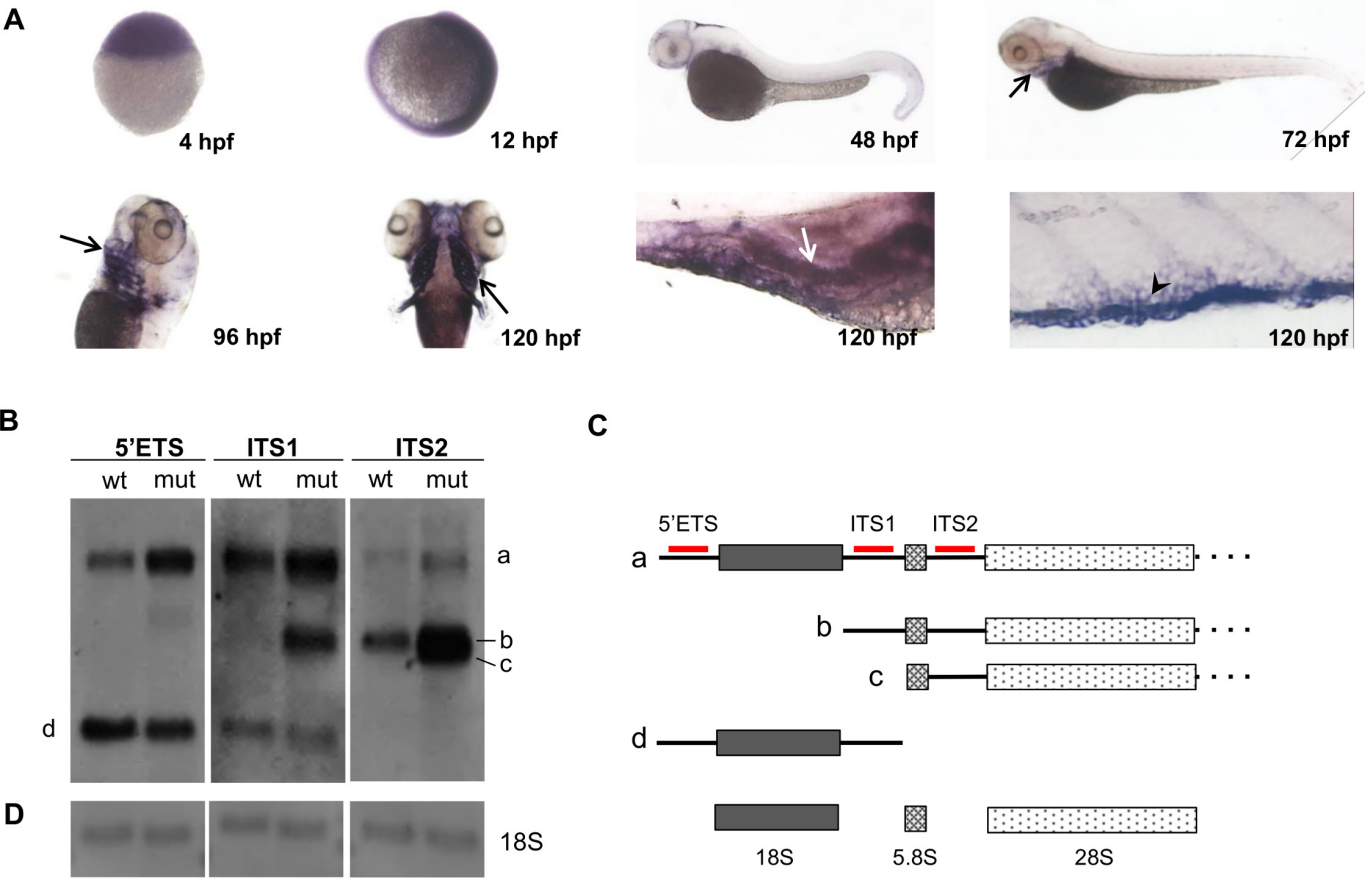
Loss-of-function *nol9* mutation leads to a defect in ITS2 pre-rRNA processing. (A) Expression pattern of *nol9* by WISH at sphere stage (4 hpf), 12 hpf, 48 hpf, 72 hpf, 96 hpf and 120 hpf. Black arrows indicate branchial arches, white arrow indicates pancreas and arrowhead indicates *nol9*-expressing cells in the CHT. (B) Representative Northern blot analysis of RNA isolated from 5 dpf *nol9* ^*sa102/sa10222*^ mutants and control (*nol9* ^*+/+*^ and *nol9* ^*+/sa1022*^) siblings using 5’ETS, ITS1 and ITS2 probes to detect rRNA processing intermediates. Corresponding rRNA intermediates (a, b, c, d) are indicated in B) and C). (C) Schematic representation of the rRNA intermediates detected in the Northern blot analysis. The sites of hybridization of the 5’ETS, ITS1 and ITS2 probes are indicated in red. (D) Methylene blue staining of the membrane was used to control for equal loading of RNA. wt–*nol9* ^*+/+*^/*nol9* ^*+/sa1022*^, mut–*nol9* ^*sa1022/sa1022*^.

Although ribosomopathies are characterized by mutations in components of a fundamental process in all cells, the clinical manifestations vary and display tissue specificity. One of the hypotheses that has been proposed to explain this tissue specificity is the distinct expression pattern of genes involved in ribosomal biogenesis [31]. We therefore assessed the expression of *nol9* during early zebrafish development using whole-mount *in situ* hybridization (WISH) (Fig 2A). *nol9* is ubiquitously expressed at 4- and 12 hpf but its expression is not apparent at 48- hpf (Fig 2A). At 72 hpf, the expression of *nol9* is evident only in the branchial arches. At 96 hpf, *nol9* expression is restricted to the branchial arches and pancreas. Finally, at 120 hpf, strong expression of *nol9* is present in the branchial arches, liver, pancreas, as well as the CHT, which is consistent with the observed digestive organ and hematological defects of *nol9* ^*sa1022/sa1022*^ larvae.

### Zebrafish Nol9 is required for 28S rRNA processing

Mature 5.8S, 18S and 28S rRNAs are produced from the 45S pre-rRNA transcript by a series of enzymatic cleavage steps (Fig 2B). Human NOL9 protein (like its homolog Grc3 in *S*. *pombe* [23]) is a polynucleotide 5’-kinase that is required for the efficient processing of the 32S precursor into 5.8S and 28S rRNAs in HeLa cells [22]. To investigate the role of Nol9 in zebrafish, we performed Northern blot analysis (Fig 2B–2D) using probes designed to hybridize to the external (5’ETS) and internal transcribed spacer (ITS1 and ITS2) regions of zebrafish 45S pre-rRNA. These probes detect the full-length rRNA precursor and all intermediate species, but not the mature rRNAs. We detected accumulation of the full-length precursor ‘a’ and of the intermediates ‘b’ and ‘c’ in *nol9* ^*sa1022/sa1022*^ mutants compared to wt siblings at 120 hpf (Fig 2B and 2C). Based on the relative signal intensities of the intermediate bands, we conclude that the block in rRNA processing in *nol9* ^*sa1022/sa1022*^ mutants occurs at the level of the intermediate ‘c’, which corresponds to the 27SB precursor in yeast and 32S in human [22, 23]. Therefore, the function of Nol9 in ITS2 processing is conserved across yeast, zebrafish and human cells [22, 23]. We hypothesize that the presence of intermediate ‘b’ reflects an alternate cleavage pathway [32]. Methylene blue staining of ribosomal RNAs after Northern transfer was used to confirm equal loading of samples (Fig 2D). Furthermore, E-bioanalyzer analysis revealed a decrease in mature 28S rRNA in *nol9* ^*sa1022/sa1022*^ larvae compared with *nol9* ^*+/+*^ *nol9* ^*+/sa1022*^ siblings at 120 hpf, while mature 18S rRNA was unaffected (S3A and S3B Fig).

### *nol9* is essential for development of zebrafish pancreatic acinar and duct cells

**Fig 3:**
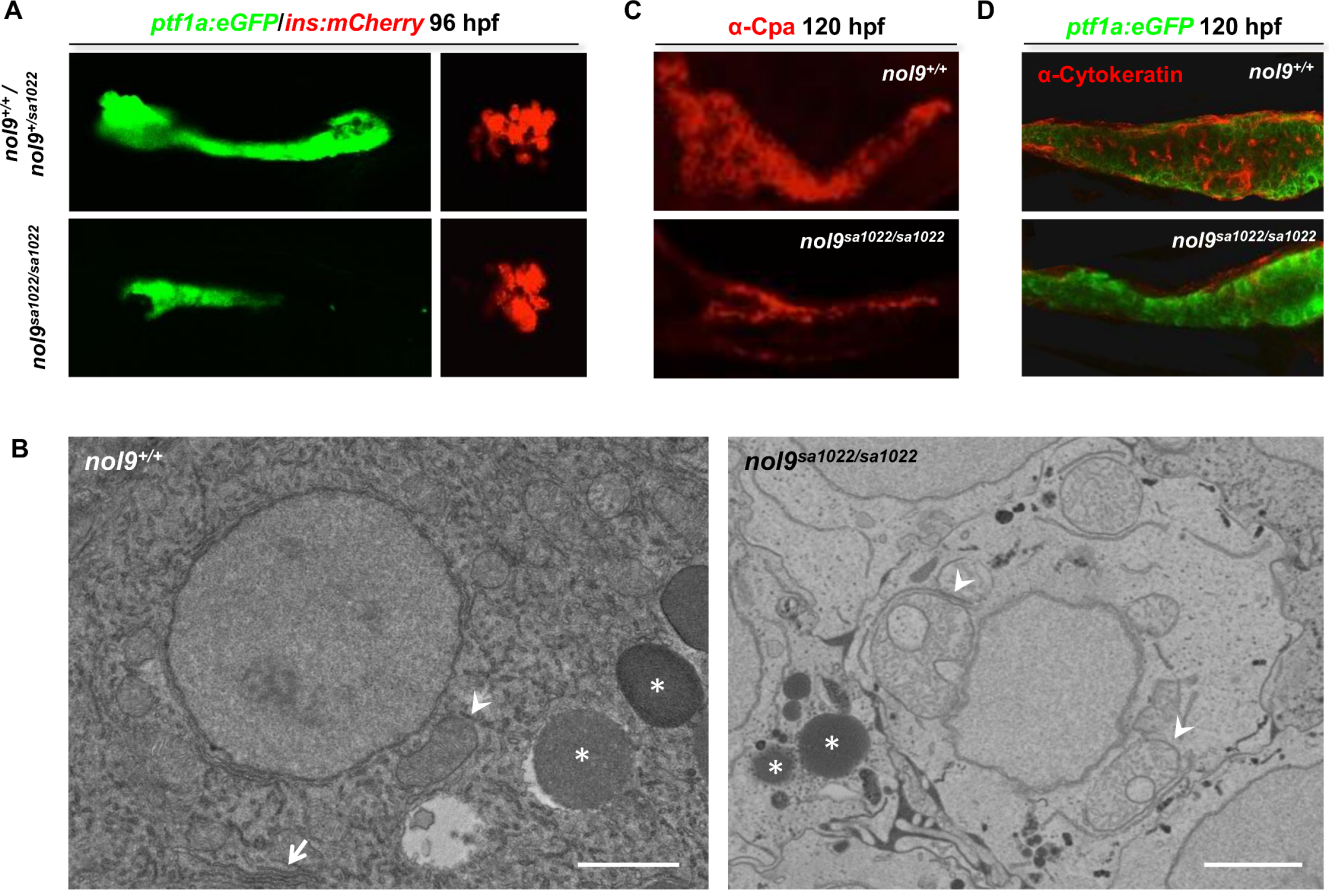
*nol9* mutation affects the development of the exocrine but not the endocrine pancreas. (A) Representative single channel confocal images of the pancreas of 96 hpf *Tg(ptf1a*:*EGFP;ins*:*mCherry)* larvae. *nol9* ^*sa1022/sa1022*^ larvae were characterized by smaller *ptf1a*-positive area (green) and similar *ins*-positive area (red) compared to *nol9* ^*+/+*^ siblings. (B) TEM pictures of the exocrine pancreas of *nol9* ^*+/+*^ and *nol9* ^*sa1022/sa1022*^ larvae at 120 hpf. The white arrow denotes endoplasmic reticulum in the *nol9* ^*+/+*^ cell. Asterisks indicate zymogen granules. Arrowheads indicate mitochondria. Scale bar: 2 μm. (C-D) Confocal images of the pancreas of *Tg(ptf1a*:*EGFP)* larvae subjected to immunohistochemistry against α-Carboxypeptidase-a (α-Cpa) (C) and α-Cytokeratin (D) at 120 hpf. (C) The exocrine pancreas differentiation marker α-Cpa (red) was detected in *nol9* ^*+/+*^ and *nol9* ^*sa1022/sa1022*^ siblings. (D) The pancreatic ducts expressing α-Cytokeratin (red) are not apparent in *nol9* ^*sa1022/sa1022*^ mutants, in contrast to the ductal network clearly visible in *nol9* ^*+/+*^ siblings.

During pancreas development, contiguous areas of the gut bud sequentially. The first posterodorsal bud develops into the endocrine principal islet whereas the second anteroventral bud gives rise to exocrine tissue, pancreatic ducts and late-forming endocrine cells [33, 34]. To dissect the role of *nol9* in pancreatic cell lineage development, we generated *nol9* ^*sa1022/sa1022*^ zebrafish in a *Tg(ins*:*mCherry)* ^*jh2*^/*Tg(ptf1a*:*EGFP)* ^*jh1*^ background [35, 36]. This allowed us to visualize *ins*:*mCherry* ^*jh2*^-expressing endocrine β-cells in conjunction with *ptf1a*:*EGFP* ^*jh1*^-expressing exocrine pancreatic cells in *nol9* ^*sa1022/sa1022*^ embryos and their wt siblings. In both wt and *nol9* ^*sa1022/sa1022*^ larvae, β-cells localized appropriately and maintained the normal islet volume from 48- to 120 hpf (Figs 3A and S4A). Furthermore, the volume of somatostatin-producing δ-cells and glucagon-producing α-cells was comparable between *nol9* ^*sa1022/sa1022*^ mutants and wt siblings at 96 hpf (S4B Fig). Thus, our data strongly suggest that the development of the endocrine pancreatic islet in *nol9* ^*sa1022/sa1022*^ larvae is normal.

In contrast, the *ptf1a*-positive exocrine pancreas appeared smaller in *nol9* ^*sa1022/sa1022*^ larvae compared to wt siblings after 72 hpf (Figs 3A and S4A and S4C). This phenotype was recapitulated by microinjection of one-cell stage zebrafish embryos with the translation blocking morpholino oligonucleotide targeted to *nol9* mRNA (S5 Fig). Transmission electron microscopy (TEM) analysis revealed that exocrine pancreatic cells in *nol9* ^*sa1022/sa1022*^ larvae lacked rough endoplasmic reticulum (RER) and had fewer and smaller zymogen granules compared to wt siblings (Fig 3B). This was in line with the observed decrease in overall protein synthesis in *nol9* ^*sa1022/sa1022*^ mutants compared to wt siblings at 120 hpf, as assessed by puromycin incorporation assay (S6 Fig). In addition, the mitochondria in exocrine pancreatic cells appeared enlarged and abnormal in *nol9* ^*sa1022/sa1022*^ mutants compared to wt siblings (Fig 3B). However, carboxypeptidase-a (α-Cpa), an exocrine pancreatic enzyme [37], was detected by immunohistochemistry in both *nol9* ^*sa1022/sa1022*^ mutants and wt siblings at 120 hpf (Fig 3C). This suggests that even though the morphology of acinar cells is severely affected in *nol9* ^*sa1022/sa1022*^ mutants, the differentiation of exocrine pancreatic cells into acinar cells is normal.

The pancreatic ducts and secondary islets are also derived from the anteroventral bud, therefore we wished to examine if their formation was affected by the loss-of-function mutation in *nol9*. Immunohistochemistry against α-cytokeratin, a marker of pancreatic ductular epithelia [38], revealed that *nol9* ^*sa1022/sa1022*^ larvae failed to form pancreatic ducts (Fig 3D) thus mirroring the defective morphogenesis of *ptf1a*-positive progenitors. Finally, we investigated secondary islet formation in *nol9* ^*sa1022/sa1022*^ larvae. The secondary islets arise from Notch-responsive progenitor cells that reside in the main pancreatic duct [39, 40]. Since the number of *ins*:*mCherry* ^*jh2*^-expressing β-cells of the secondary islets is scarce in wt larvae at 120 hpf, we utilized Notch inhibition as a way to accelerate differentiation of the endocrine cells of the secondary islets [39]. We incubated *nol9* ^*sa1022/sa1022*^ embryos and their wt siblings in either 100 μM of Notch inhibitor *N*-[*N*-(3,5-difluorophenacetyl)-L-alanyl]-*S*-phenylglycine *t*-butyl ester (DAPT) or dimethyl sulfoxide (DMSO), as a control, from 72 to 120 hpf. The DAPT treatment resulted in a comparable increase in the percentage of both *nol9* ^*sa1022/sa1022*^ larvae and wt siblings displaying *ins*:*mCherry* ^*jh2*^-expressing secondary islets compared to DMSO treated embryos (S7A and S7B Fig). Our observations suggest that the relative number of secondary islet progenitor cells is retained even in the absence of Nol9. Overall we conclude that the defects arising from the loss-of-function *nol9* mutation affect only cells of the exocrine pancreas, namely the acinar and pancreatic duct cells.

### *nol9* ^*sa1022/sa1022*^ embryos have impaired proliferation of exocrine pancreatic cells

The reduced size of the exocrine pancreas in *nol9* ^*sa1022/sa1022*^ larvae at 96 hpf could be attributed to increased apoptosis and/or impaired cell proliferation. The terminal deoxynucleotidyl transferase dUTP nick end labelling (TUNEL) assay revealed apoptotic cells in different areas of the zebrafish, including the tail, but none in the pancreas of either *nol9* ^*sa1022/sa1022*^ or their wt siblings at 96 hpf (S7C and S7D Fig). These data indicate that apoptosis does not contribute to the exocrine pancreatic defect in *nol9* ^*sa1022/sa1022*^ mutants. In order to examine cell proliferation in the pancreas of *nol9* ^*sa1022/sa1022*^ larvae, we measured 5-bromo-2’-deoxyuridine (BrdU) incorporation in 96 hpf larvae from a *Tg(ptf1a*:*EGFP)/nol9* ^*+/sa1022*^ x *Tg(ptf1a*:*EGFP)/nol9* ^*+/sa1022*^ cross. We detected fewer *ptf1a*-expressing cells in S phase after normalising for pancreatic volume in *nol9* ^*sa1022/sa1022*^ mutants compared to siblings control (S8A and S8B Fig). Thus, the impaired expansion of the pancreas in *nol9* ^*sa1022/sa1022*^ mutants most likely results from decreased cell proliferation of pancreatic progenitor cells.

### Loss of *nol9* function reduces the proliferation of HSPCs and disrupts the cellular architecture of the CHT

**Fig 4:**
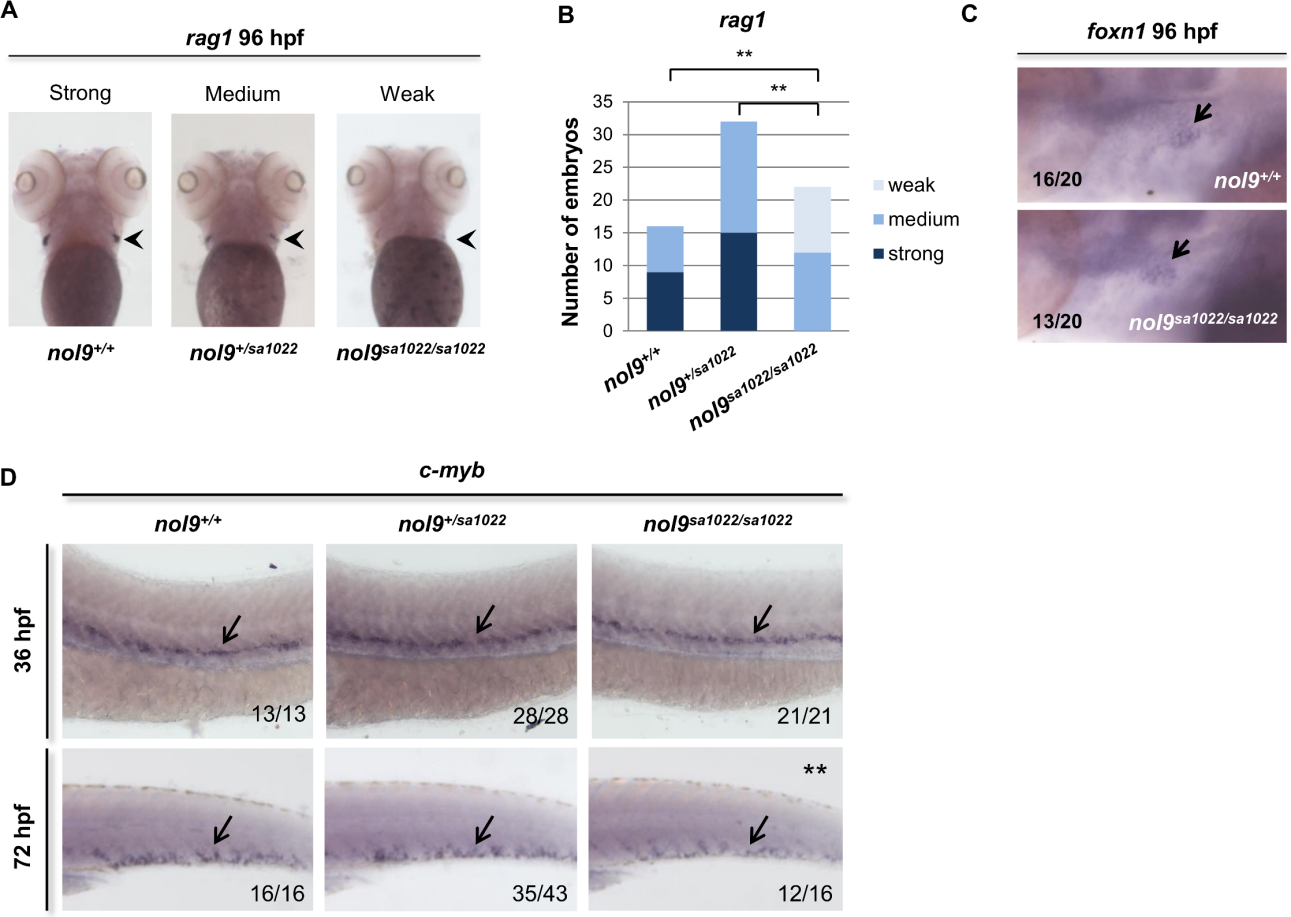
*nol9* ^*sa1022/sa1022*^ mutants show a decrease in the number of lymphocytes and HSPCs. A) Whole-mount *in situ* hybridization with lymphocyte-specific *rag1* probe (arrowhead) revealed that *nol9* ^*sa1022/sa1022*^ larvae displayed weak to medium *rag1* expression at 96 hpf, compared to medium to strong signal in *nol9* ^*+/+*^ or *nol9* ^*+/sa1022*^ siblings. Larvae are oriented anterior to the top and ventral up. (B) The number of larvae displaying different degrees of *rag1* expression as assessed by WISH. Data are represented as the number of larvae belonging to each phenotypic group. Fisher’s exact test, ** p<0.01. (C) Whole-mount *in situ* hybridization against the thymic epithelium marker *foxn1* at 96 hpf revealed a similar level of signal (arrow) in *nol9* ^*+/+*^ and *nol9* ^*sa1022/sa1022*^ siblings. (D) Whole-mount *in situ* hybridization using a *c-myb* riboprobe was used to assess the number of HSCs emerging in the AGM region at 36 hpf (arrow) and the number of HSPCs in the CHT (arrow) at 72 hpf. Representative pictures of the AGM region (36 hpf) and the CHT (72 hpf) are shown. Mutant *nol9* ^*sa1022/sa1022*^ embryos displayed normal *c-myb* signal at 36 hpf and decreased *c-myb* signal at 72 hpf compared to wt siblings. Fisher’s exact test, **, p<0.01. All embryos are oriented with anterior to the left and dorsal to the top. Numbers represent embryos with the displayed phenotype out of the total number of embryos examined.

Hematological defects in ribosomopathies often include, but are not limited to, anemia. In Shwachman-Diamond Syndrome, the most common hematological symptom is neutropenia, followed by anemia and thrombocytopenia [41]. To dissect hematological defects caused by the loss-of-function mutation in *nol9*, we evaluated blood lineage differentiation in *nol9* ^*sa1022/sa1022*^ larvae and their heterozygous and wt siblings. Interestingly, while *nol9* was essential for definitive erythropoiesis (Fig 1C), all larvae from a *Tg(cd41*:*EGFP)/nol9* ^*+/sa1022*^ x *nol9* ^*+/sa1022*^ cross had comparable numbers of *cd41* ^high^ thrombocytes at 96 hpf (S9A and S9B Fig). Since thrombocytes are the ontogenetically closest lineage to definitive erythrocytes, these data suggest that the thrombocyte-erythroid progenitors are not affected in *nol9* ^*sa1022/sa1022*^ fish. Similarly, we did not observe a significant difference in the number of neutrophils between *nol9* ^*sa1022/sa1022*^ larvae and their wt siblings at 72 hpf, as assessed by Sudan Black staining (S9C and S9D Fig). In contrast, *in situ* hybridization using *rag1* riboprobe revealed that the number of lymphocytes in *nol9* ^*sa1022/sa1022*^ larvae was markedly decreased compared to heterozygous and wt siblings at 96 hpf (Fig 4A and 4B). However, *nol9* ^*sa1022/sa1022*^ mutants and wt siblings showed comparable levels of expression of the thymic epithelial marker *foxn1* [42], as assessed by *in situ* hybridization at 96 hpf (Fig 4C). These data suggest that in *nol9* ^*sa1022/sa1022*^ mutants the development of the thymus is unaffected compared to wt siblings and that the observed decrease in the number of lymphocytes is not secondary to a defect of the thymic niche.

**Fig 5:**
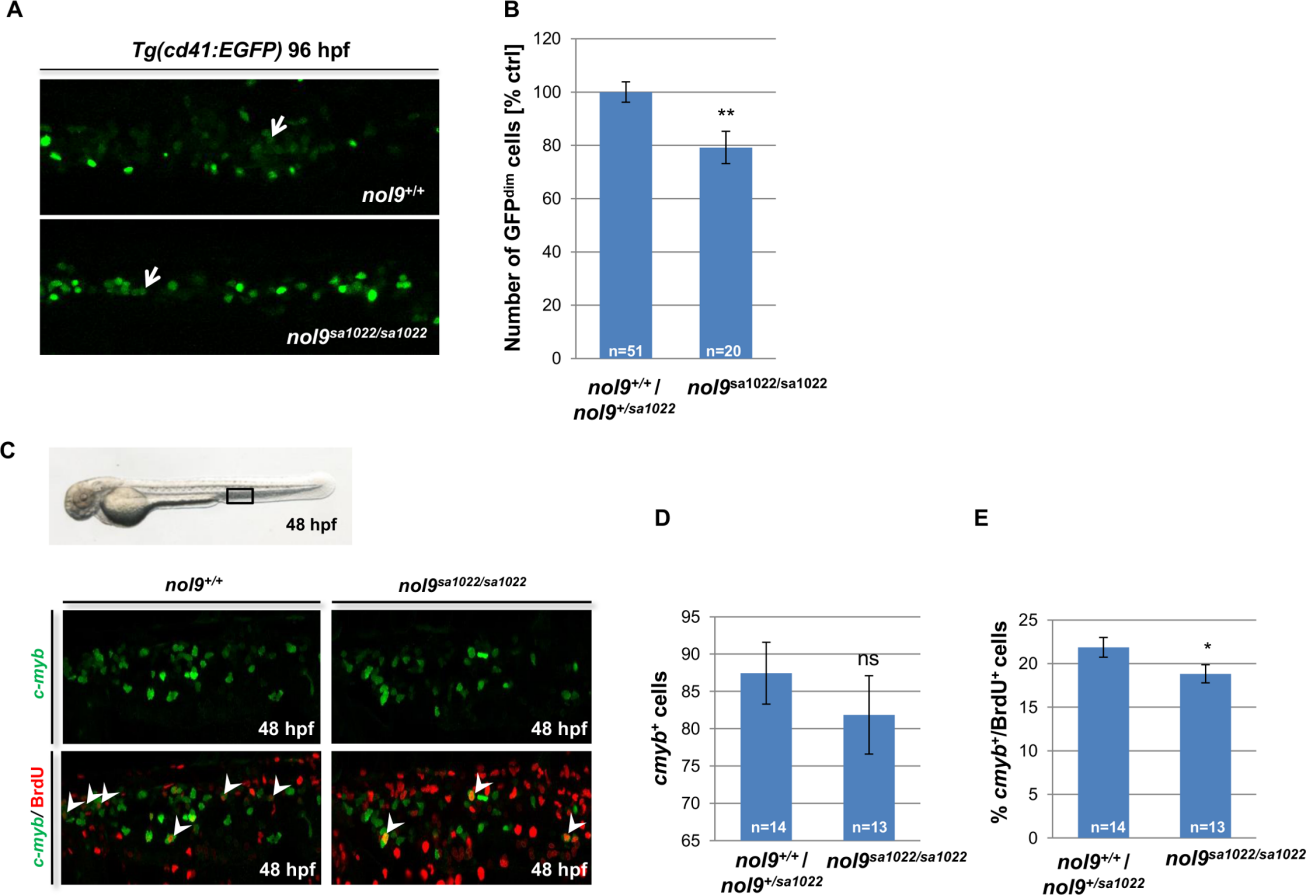
*nol9* ^*sa1022/sa1022*^ mutants show a decrease in proliferation of *c-myb* ^*+*^ cells. (A) Representative maximum projection confocal images showing HSPCs (GFP^dim^, arrow) in the CHT of *Tg(cd41*:*EGFP)* larvae at 96 hpf. Larvae are oriented with anterior to the left and dorsal to the top. (B) The number of GFP^dim^ HSPCs observed in the CHT region of *nol9* ^*sa1022/sa1022*^ mutants (n = 20) and their wt siblings (n = 51) at 96 hpf. Data are represented as the mean +/- SEM, Student’s *t*-test, **, p<0.01. (C) Representative confocal images of the CHT of *Tg(cmyb*:*EGFP)* embryos subjected to BrdU incorporation assay at 48 hpf. Arrowheads mark double positive BrdU^+^ *c-myb* ^+^ cells. Bright field image of a 48 hpf embryo shows the imaged part of the CHT. (D) The total number of *c-myb* ^*+*^ cells observed in the CHT of *nol9* ^*sa1022/sa1022*^ embryos (n = 13) and their wt siblings (n = 14) subjected to the BrdU incorporation assay at 48 hpf. Data are represented as the mean +/- SEM. Student’s *t*-test. ns—not significant. (E) The percentage of BrdU^+^ cells within the *c-myb* ^*+*^ population in the CHT of *nol9* ^*sa1022/sa1022*^ embryos (n = 13) and their wt siblings (n = 14) subjected to the BrdU incorporation assay at 48 hpf. Data are represented as the mean +/- SEM, Student’s *t*-test, *, p<0.05.

To further investigate the mechanism behind defects in hematopoiesis in *nol9* ^*sa1022/sa1022*^ embryos, we assessed the temporal expression of one of the early markers of hematopoietic stem cells (HSCs), the transcriptional activator *c-myb* [43]. *In situ* hybridization using *c-myb* riboprobe revealed that the specification of HSCs in the aorta–gonad–mesonephros (AGM) was normal in *nol9* ^*sa1022/sa1022*^ embryos at 36 hpf (Fig 4D). However, the number of hematopoietic stem and progenitor cells (HSPCs) was markedly decreased in the CHT at 72 hpf (Fig 4D). These data were further corroborated by flow cytometric analysis of *c-myb* ^+^ cells in wt and *nol9* ^*sa1022/sa1022*^ larvae at 96 hpf. We detected a significant decrease in the number of EGFP^+^ cells sorted from the dissected CHT region of *Tg(c-myb*:*EGFP)/nol9* ^*sa1022/sa1022*^ compared to *Tg(c-myb*:*EGFP)/nol9* ^*+/+*^ larvae (S10A–S10C Fig). Quantification of HSPCs using the *Tg(cd41*:*EGFP)* line [44] confirmed a decrease in the number of *cd41* ^*low*^ (GFP^dim^) HSPCs in the CHT of *nol9* ^*sa1022/sa1022*^ mutants compared to wt siblings at 96 hpf (Fig 5A and 5B). Similar to our earlier observations in the pancreas, the reduced number of *c-myb* ^*+*^ cells was not due to increased apoptosis in the CHT region of *nol9* ^*sa1022/sa1022*^ larvae (S11 Fig). Instead, 5-bromo-2'-deoxyuridine (BrdU) incorporation assay revealed that the proliferation of HSPCs was significantly decreased in *nol9* ^*sa1022/sa1022*^ embryos at 48 hpf (Fig 5C–5E). This led us to conclude that the decrease in the number of HSPCs in *nol9* ^*sa1022/sa1022*^ larvae at 72 hpf is due to a defect in HSPC proliferation and not due to increased apoptosis.

**Fig 6:**
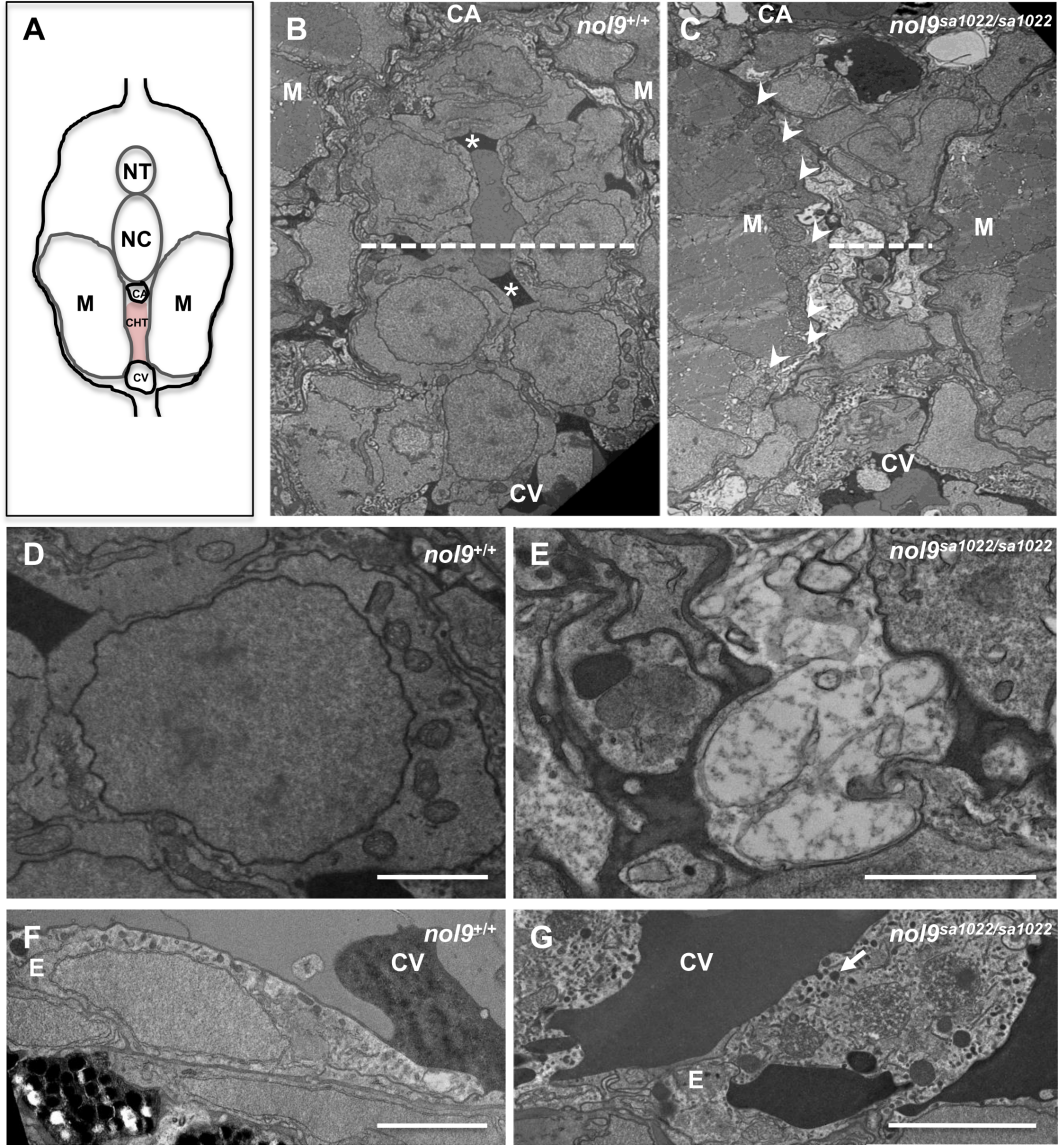
Ultrastructural studies of caudal hematopoietic tissue (CHT) in *nol9* ^*sa1022/sa1022*^ larvae. (A) Schematic representation of a transversal section in the CHT region of a 120 hpf zebrafish, dorsal up. NT–neural tube, NC–notochord, M—myotome, CA–caudal artery, CHT–caudal hematopoietic tissue, CV–caudal vein. (B-C) A low-magnification TEM image of the CHT from a *nol9* ^*+/+*^ (B, n = 2) and *nol9* ^*sa1022/sa1022*^ (C, n = 2) larvae. Dashed line denotes the width of the CHT. Arrowheads denote mitochondrial profiles visible within myotomes (M). Asterisks denote extracellular matrix (ECM). (D-E) High magnification TEM image of cells present in the CHT of *nol9* ^*+/+*^ (D) and *nol9* ^*sa1022/sa1022*^ (E) larvae. (F-G) High magnification TEM image of endothelial cells in the caudal vein of *nol9* ^*+/+*^ (F) and *nol9* ^*sa1022/sa1022*^ (G) larvae. Arrow denotes vesicles visible in the cytoplasm of the endothelial cell, reminiscent of lipofuscin. E–endothelium. Scale bars: 2 μm.

Although the gross morphology of the CHT and vessels appeared normal in *nol9* ^*sa1022/sa1022*^ mutants, we wanted to examine it further at the ultrastructural level. To this end we used transmission electron microscopy (TEM) to assess the cellular architecture of the CHT (Fig 6). At 120 hpf, CHT is confined within the space limited dorsally by the caudal artery, ventrally by the caudal vein, and laterally by myotomes (Fig 6A). In wt fish the CHT contained a number of rounded cells supported by deposits of extracellular matrix (ECM) (Fig 6D). In contrast, in *nol9* ^*sa1022/sa1022*^ larvae the CHT area was largely depleted of cells and ECM and instead filled by electron-loose, possibly necrotic, cell projections full of debris (Fig 6E). As a result, the CHT area was markedly decreased in *nol9* ^*sa1022/sa1022*^ mutants compared to wt siblings (Fig 6B and 6C). Interestingly, we also observed signs of degeneration of endothelial cells in the caudal vein of *nol9* ^*sa1022/sa1022*^ mutants. Endothelial cells seemed “swollen” and contained a high number of, what appeared to be, lipofuscin granules (Fig 6F and 6G). The accumulation of lipofuscin granules is often associated with damage to mitochondria, membrane and lysosomes and is implicated in many degenerative diseases [45]. Thus, our TEM analysis further underlines the complexity of the phenotype observed in *nol9* ^*sa1022/sa1022*^ larvae and suggests much broader changes in the CHT niche, including ECM and endothelial cells.

### The pancreatic and hematological defects in *nol9* ^*sa1022/sa1022*^ larvae are mTOR independent

Sufficient protein synthesis is essential for cell growth and proliferation and one of the pathways implicated in the regulation of these processes is the Target of Rapamycin (TOR) pathway. The TOR pathway controls ribosomal protein gene transcription and rRNA synthesis and processing [46, 47]. To assess whether the defective protein synthesis in *nol9* ^*sa1022/sa1022*^ larvae activates the TOR pathway, we assessed the phosphorylation of the mTOR target 4E-binding protein 1 (p4EBP1) in *nol9* ^*sa1022/sa1022*^ and wt larvae. We found no difference in the levels of phosphorylated p4EBP1 in *nol9* ^*sa1022/sa1022*^ larvae compared to *nol9* ^*+/+*^ and *nol9* ^*+/sa1022*^ siblings at 120 hpf using Western blot analysis (S12A Fig). In addition, we tested the effect of L-Leucine on exocrine pancreas and hematopoietic phenotypes in *nol9* ^*sa1022/sa1022*^ mutants. L-Leucine was previously reported to improve the defects resulting from faulty ribosome biogenesis by activating mRNA translation via the mTOR pathway [17, 48]. In *nol9* ^*sa1022/sa1022*^ mutants, however, the mean volume of the *ptf1a*-expressing region remained significantly smaller when compared to *nol9* ^*+/+*^ or *nol9* ^*sa1022/+*^ larvae regardless of the treatment of embryos with L-Leucine or its inactive enantiomer D-Leucine (S12B and S12C Fig). Similarly, *in situ* hybridization against *hbae1* revealed that *nol9* ^*sa1022/sa1022*^ larvae had significantly lower expression of *hbae1* than their *nol9* ^*+/+*^ or *nol9* ^*sa1022/+*^ siblings at 120 hpf, irrespective of whether they were treated with L-Leucine, D-Leucine or L-Alanine (S12D Fig). Therefore, we concluded that both the exocrine pancreas and hematopoietic phenotypes observed in *nol9* ^*sa1022/sa1022*^ mutants are independent of the mTOR pathway. We do not, however, rule out the possibility that defects in translation of specific mRNAs may occur independently of mTOR and may directly contribute to the observed phenotype.

### The role of *tp53* in hematopoiesis and pancreas morphogenesis in *nol9* ^*sa1022/sa1022*^ larvae

Studies have shown that Tp53-dependent mechanisms contribute to the phenotypes resulting from defective ribosome biogenesis [6–8, 11–13, 15, 16, 49–53]. Publicly available mRNA expression data generated by the Zebrafish Mutation Project showed that on a whole-organism level, *tp53* mRNA is 4.3-fold more abundant in *nol9* ^*sa1022/sa1022*^ mutants than in their wt siblings at 120 hpf (adjusted p-value = 6.49 x 10^−79^) [54]. In order to investigate whether the exocrine pancreas phenotype of *nol9* ^*sa1022/sa1022*^ larvae is dependent on Tp53 signalling, we outcrossed the *Tg(ins*:*mCherry)* ^*jh2*^ */Tg(ptf1a*:*EGFP)* ^*jh1*^/*nol9* ^*+/sa1022*^ line to the *tp53* ^*+/zdf1*^ line [55]. In *tp53* ^*/zdf1/zdf1*^ mutant fish, the M214K missense mutation interferes with Tp53 activity by disrupting its DNA-binding domain. We found that at 120 hpf, the mean volume of the *ptf1a*-expressing region of *nol9* ^*sa1022/sa1022*^ */tp53* ^*+/+*^ larvae was not significantly different from that of *nol9* ^*sa1022/sa1022*^ */tp53* ^*zdf1/zdf1*^ larvae although both were significantly smaller than the mean volume of the *ptf1a*-expressing region of *nol9* ^*+/+*^ */tp53* ^*+/+*^ and *nol9* ^*+/+*^ */tp53* ^*zdf1/zdf1*^ larvae (S13A and S13B Fig). These data suggest that the exocrine pancreas phenotype of *nol9* ^*sa1022/sa1022*^ is independent of Tp53 signalling.

**Fig 7:**
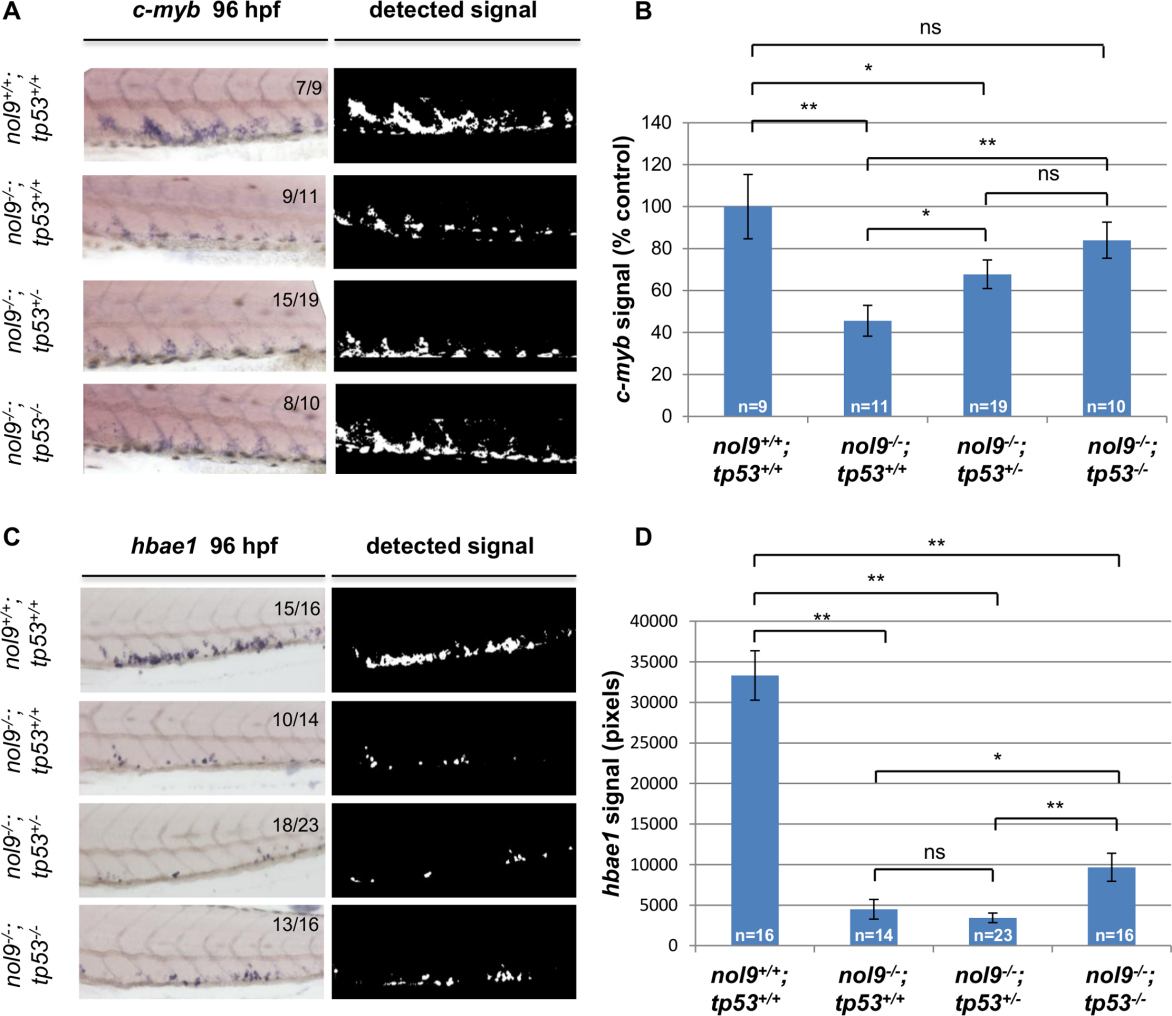
The number of HSPCs is rescued in a *tp53* mutant background. (A) The CHT of 96 hpf larvae from a *nol9* ^*+/sa1022*^ *;tp53* ^*+/zdf1*^ x *nol9* ^*+/sa1022*^ *;tp53* ^*+/zdf1*^ cross stained by WISH against *c-myb*. Signal extracted from the corresponding WISH photograph is shown. Numbers represent larvae with the displayed phenotype out of the total number of larvae examined. (B) Quantification of *c-myb in situ* signal for 96 hpf larvae from a *nol9* ^*+/sa1022*^ *;tp53* ^*+/zdf1*^ x *nol9* ^*+/sa1022*^ *;tp53* ^*+/zdf1*^ cross, depending on their genotype. Data are represented as the mean number of pixels +/- SEM. *nol9* ^*+/+*^ *;tp53* ^*+/+*^ n = 9; *nol9* ^*-/-*^ *;tp53* ^*+/+*^ n = 11; *nol9* ^*-/-*^ *;tp53* ^*+/zdf1*^ n = 19; *nol9* ^*-/-*^ *; tp53* ^*zdf1/zdf1*^ n = 10. Two-tailed Student’s *t*-Test, *, p<0.05; **, p<0.01. (C) Representative pictures of the CHT of 96 hpf larvae from a *nol9* ^*+/sa1022*^ *;tp53* ^*+/zdf1*^ x *nol9* ^*+/sa1022*^ *;tp53* ^*+/zdf1*^ cross stained by WISH against *hbae1*. Signal extracted from the corresponding WISH photograph is shown. (D) Quantification of *hbae1* WISH signal for larvae from a *nol9* ^*+/sa1022*^ *;tp53* ^*+/zdf1*^ x *nol9* ^*+/sa1022*^ *;tp53* ^*+/zdf1*^ cross, depending on their genotype. Data are represented as the mean number of pixels +/- SEM. *nol9* ^*+/+*^ *;tp53* ^*+/+*^ n = 16; *nol9* ^*-/-*^ *;tp53* ^*+/+*^ n = 14; *nol9* ^*-/-*^ *;tp53* ^*+/-*^ n = 23; *nol9* ^*-/-*^ *;tp53* ^*-/-*^ n = 16. Two-tailed Student’s *t*-Test, *, p<0.05; **, p<0.01. Within the figure, *nol9* ^*sa1022*^ allele has been denoted as *nol9* ^*-*^ and *tp53* ^*zdf1*^ as *tp53* ^*-*^.

In order to assess whether the hematopoietic defects observed in *nol9* ^*sa1022/sa1022*^ mutants are Tp53-dependent, we in-crossed *nol9* ^*+/sa1022*^ */tp53* ^*zdf1/+*^ fish and used their progeny for *in situ* hybridization against *c-myb* and *hbae1* at 96 hpf (Fig 7A–7D). Statistical analysis revealed that in the *tp53* ^*zdf1/zdf1*^ background, the level of *c-myb* signal in *nol9* ^*sa1022/sa1022*^ larvae reverted to wt (*nol9* ^*+/+*^;*p53* ^*+/+*^) levels (Fig 7A and 7B). In contrast, although *nol9* ^*sa1022/sa1022*^ */tp53* ^*zdf1/zdf1*^ larvae had a significant increase in the *hbae1 in situ* signal compared to *tp53* ^*+/zdf1*^ and *tp53* ^*+/+*^ siblings, the *hbae1* signal was still over three-times lower than in wt (*nol9* ^*+/+*^/*p53* ^*+/+*^) siblings (Fig 7C and 7D). Therefore, we conclude that in *nol9* ^*sa1022/sa1022*^ mutants the defect in the proliferation of HSPCs, but not in the differentiation of definitive erythrocytes, is Tp53-dependent.

**Fig 8:**
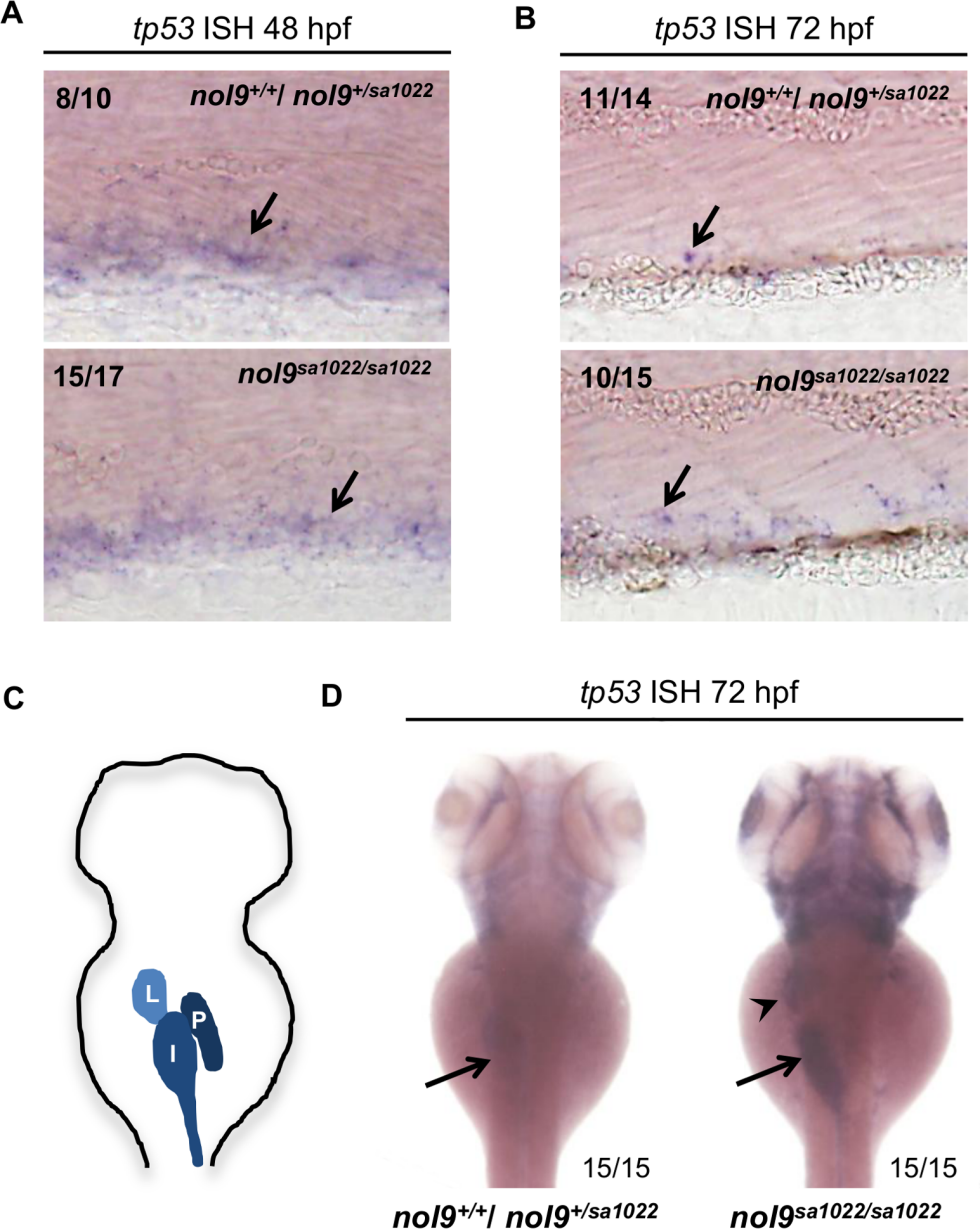
*nol9* ^*sa1022/sa1022*^ embryos display tissue-specific upregulation of *tp53*. Representative images of embryos stained by whole-mount *in situ* hybridization against *tp53*. (A) At 48 hpf, similar levels of *tp53* signal (arrow) was detected in the CHT of *nol9* ^*sa1022/sa1022*^ embryos and their wild-type siblings. (B) At 72 hpf, *nol9* ^*sa1022/sa1022*^ mutant embryos were characterized by more *tp53* signal in the CHT than their wt siblings. Mann-Whitney U test, p<0.05. (A-B) All embryos are oriented with anterior to the left and dorsal to the top. (C) Schematic representation of digestive organs in a wild-type 72 hpf zebrafish larva. I–intestine, L- liver, P- pancreas. (D) At 72 hpf, *nol9* ^*sa1022/sa1022*^ mutant embryos display strong *tp53* signal in the liver (arrowhead) and intestine (arrow), compared to weak signal in the intestine of wild-type siblings. (C-D) Dorsal view anterior up.

To further investigate the involvement of Tp53 in the development of defects in different tissues of *nol9* ^*sa1022/sa1022*^ mutants, we assessed the levels of *tp53* mRNA in *nol9* ^*sa1022/sa1022*^ mutants and wt siblings by whole-mount *in situ* hybridization (Fig 8). At 48 hpf, *tp53* was expressed at high levels in the CHT of *nol9* ^*sa1022/sa1022*^ mutants and wt siblings alike (Fig 8A). However, at 72 hpf the expression of *tp53* remained high in the CHT of *nol9* ^*sa1022/sa1022*^ mutants but was down-regulated in wt siblings (Fig 8B). At this stage, *tp53* was also significantly up-regulated in the liver and intestine of *nol9* ^*sa1022/sa1022*^ mutants, but *tp53* expression was not detected in the pancreas of *nol9* ^*sa1022/sa1022*^ mutants nor wt siblings (Fig 8C and 8D). In conclusion, expression of *tp53* was detected in the CHT but not in the pancreas prior to the development of the phenotype in *nol9* ^*sa1022/sa1022*^ mutants, i.e. 48 hpf for the CHT and 72 hpf for the pancreas.

## Discussion

In this study, we have characterized the phenotype of the loss-of-function *nol9* ^*sa1022/sa1022*^ zebrafish mutant. We have shown that in zebrafish, similar to human, Nol9 is involved in 28S rRNA processing. In addition, *nol9* ^*sa1022/sa1022*^ larvae had impaired development of the intestine, liver, pancreas and definitive erythrocytes and lymphocytes, thus recapitulating several common features of ribosomopathies in human.

Although ribosomopathies have a wide spectrum of clinical manifestations, many are characterized by hypoproliferative phenotypes. Zebrafish harboring a loss-of-function *nol9* ^*sa1022*^ mutation displayed an early hypoproliferative defect restricted to progenitor cells of the digestive and hematopoietic systems. Both *ptf1a*-positive progenitor cells of the exocrine pancreas and *cmyb*-positive HSPCs had significantly lower rates of proliferation in *nol9* ^*sa1022/sa1022*^ larvae compared to their wt siblings. This tissue specific phenotype can be partially explained by the distinct expression pattern of *nol9*. Indeed, we observed that *nol9* was most highly expressed in branchial arches, liver, pancreas, as well as caudal hematopoietic tissue at 120 hpf. Alternatively, Nol9 may have tissue-specific interacting partners and extra-ribosomal functions in the digestive organs and in HSPCs [56, 57]. Furthermore, it has been proposed that the spectrum of translated mRNAs might change with the overall reduction of fully functional cytoplasmic ribosomes or specific ribosomal biogenesis proteins, suggesting that ribosome composition may vary between distinct cell types [56–58]. Another explanation for the tissue specific effect of *nol9* depletion stems from the high proliferative rate of the affected tissues. The reason for this is twofold. Firstly, it is reasonable to speculate, that highly proliferative cell populations, such as pancreatic cells and HSPCs, deplete maternally-derived Nol9 protein earlier than other cell populations [58]. And secondly, defects in ribosomal biogenesis affect primarily highly proliferative cells due to their high demand for ribosome production. This is particularly germane to HSCs which are shown to be very sensitive to changes in the level of protein synthesis [59].

Impaired development of the pancreas, liver and intestine has been a recurrent pathological feature in many zebrafish models of ribosomopathies, including *nil per os* (*npo*) [60], *digestive expansion factor* (*def*) [8], *titania* (*tti*) [61] and *nucleolar protein with MIF4G domain 1* (*nom1*) [62]. In contrast, the hematopoietic defects have been studied to a lesser extent; the reported phenotypes are usually limited to primitive erythropoiesis or specification of HSCs as in *rpl11*, *rps19*, *rps7*, *rps29* and *nop10* mutants [16, 18, 53, 63, 64]. In our *nol9* ^*sa1022/sa1022*^ model, primitive erythropoiesis, as well as specification of HSCs, was normal. However, the *nol9* ^*sa1022/sa1022*^ larvae had decreased numbers of definitive erythroid and lymphoid cells, whereas the number of thrombocytes remained within the normal range at 96 hpf. Thus, to the best of our knowledge, the *nol9* ^*sa1022/sa1022*^ mutant is the only zebrafish model of ribosomopathy in which the later stages of definitive hematopoiesis have been described. Although blood defects were initially restricted to specific lineages, the whole hematopoietic tissue appeared degenerated at 120 hpf. The CHT of the *nol9* ^*sa1022/sa1022*^ larvae was largely depleted of cells and ECM and filled with a number of cell projections that were reminiscent of cells undergoing necrosis. Interestingly, we also detected signs of pathological processes in endothelial cells of the caudal vein. The endothelial cells appeared swollen and filled with lipofuscin granules, featuring structural changes not previously reported in other zebrafish models of ribosomopathy. Therefore, the phenotype of the *nol9* ^*sa1022/sa1022*^ mutants should be viewed as a progressive degenerative process, which culminates with the death of larvae by 10 dpf.

It is hypothesized that impaired ribosomal biogenesis promotes excess free ribosomal proteins in the nucleus, resulting in nucleolar stress. In response to this stress, free ribosomal proteins (including RPL5, RPL11, RPL23, RPS7 and RPL26) bind and inactivate MDM2, which results in Tp53 stabilization and Tp53-dependent cell cycle arrest [65–71]. However, Tp53-independent processes involving cell cycle arrest and apoptosis due to ribosome biogenesis defects have been also described [72–75]. As in *nom1*, *tti*, *pes*, *rpl3*, *rpl23a* and *rpl6* mutants, the defect in pancreatic development in *nol9* ^*sa1022/sa1022*^ mutants was Tp53-independent [61, 62, 76, 77]. In *rpl11* mutants, defects in specification of HSCs as well as primitive erythropoiesis were Tp53-dependent [16]. Similarly, mice with heterozygous mutations in ribosomal genes *Rps20* and *Rps19* had a *tp53*-mediated decrease in the number of erythrocytes [78]. Interestingly, in our *nol9* ^*sa1022/sa1022*^ */tp53* ^*zdf1/zdf1*^ double mutant model, the number of HSPCs in the CHT was restored to wt levels, whereas the number of definitive erythrocytes was only partially rescued. This suggests that in *nol9* ^*sa1022/sa1022*^ mutants there are two independent hematopoietic defects—one affecting HSPC proliferation and the other affecting definitive erythrocyte differentiation. Interestingly, we observed high expression of *tp53* in the CHT of wt and *nol9* ^*sa1022/sa1022*^ embryos at 48 hpf, which suggests that Tp53 may play a developmental role in HSPCs before 72 hpf, which could account for the different responses of HSPCs, definitive erythrocytes and exocrine pancreas to the loss of *tp53* in *nol9* ^*sa1022/sa1022*^ mutants.

It has been speculated that disruption in translational machinery in erythroid precursors leads to anemia in many ribosomopathies [3]. For example, altered translation of the key erythroid transcription factor GATA-1 has also been reported in DBA [79]. The late stage erythroid cells exhibit rapid cell division and extensive translational demand for globin synthesis. Therefore, anemia may be attributed to the hypersensitivity of these cells to the reduced level of mRNA translation. Treatment of embryos with amino acid L-Leucine, a potent activator of the mTOR pathway and mRNA translation, has been shown to alleviate anemia in *rps14* and *rps19* morpholino knock-down zebrafish embryos [17, 80]. In *nol9* ^*sa1022/sa1022*^ mutants, L-Leucine treatment did not rescue the erythroid or pancreatic phenotype suggesting that these are independent of mTOR.

The phenotypic features of the *nol9* ^*sa1022/sa1022*^ mutants are highly reminiscent of the tissue specific clinical features of Shwachman-Diamond syndrome (SDS) that is associated with deficiency of the *SBDS* (Shwachman-Bodian-Diamond Syndrome) protein [24]. SBDS plays a role in the same process as NOL9, although at a later stage: it is required for late cytoplasmic maturation of 60S ribosomal subunits and translational activation of ribosomes [25–27]. In zebrafish, *sbds* and *nol9* are both highly expressed in the pancreas at 120 hpf [77, 81]. In addition, MO knock-down of *sbds* leads to a Tp53-independent defect in the development of the exocrine, but not endocrine pancreas [77], in line with the phenotype observed in *nol9* ^*sa1022/sa1022*^ mutants. The involvement of SBDS and NOL9 in 60S ribosomal subunit biogenesis, coupled with the remarkable similarity of the phenotypes resulting from depletion of both genes in zebrafish (some of which mimic clinical manifestations of SDS) strongly supports the view that *nol9* ^*sa1022/sa1022*^ mutant zebrafish are a highly relevant, valuable ribosomopathy model system.

Our study describes the zebrafish *nol9* ^*sa1022/sa1022*^ mutant, a novel vertebrate model of ribosomopathy, which recapitulates key human disease characteristics. Further characterisation of the *nol9* ^*sa1022/sa1022*^ mutant will foster insights into the molecular pathology of ribosomopathies and provide fundamental new insights into how ribosome dysfunction leads to tissue-specific pathologies. Ultimately, the use of good genetically tractable models, such as the zebrafish *nol9* ^*sa1022/sa1022*^ mutant, will have significant impact not only on our understanding of the range of human ribosomopathies but will also accelerate the development of targeted therapies.

## Materials and Methods

### Ethics statement

The maintenance, embryo collection and staging of the wild type (Tubingen Long Fin) and transgenic and mutant zebrafish lines (*Tg(cd41*:*EGFP)*, Tg(*c-myb*:*EGFP)*, *Tg(ptf1a*:*EGFP)*, *Tg(ins*:*mCherry)*, *tp53* ^*zdf1*^, *nol9* ^*sa1022*^) were performed in accordance with EU regulations on laboratory animals, as previously described [82, 83]. Melanization of embryos was prevented by incubating embryos with 0.002% phenylthiourea (PTU, Sigma Aldrich) from 24 hpf.

### Genotyping

DNA isolation and genotyping using allele-specific probes were performed as previously described [84].

### Whole-mount *in situ* hybridization

Whole-mount i*n situ* hybridization was performed with gene-specific probes against *c-myb*, *rag1*, *hbae1*, *prox1*, *fabp2*, *foxn1* and *tp53* as previously described [85]. Primers used for probe synthesis against *nol9* were: 5’-GACAATGAAAGTACACAAGGTTC-3’ (forward) and 5’-TAATACGACTCACTATAGGGTAACACTGCACGGTTCTTGG-3’ (reverse). The riboprobe was synthesized by in vitro transcription using T7 polymerase, with the PCR product used as the template.

Quantification of *prox1* WISH signal was performed by measuring the area of stained tissue in ImageJ.

Quantification of *hbae1* and *c-myb* WISH signal in the CHT region was performed in an objective manner by counting the number of pixels with color within a prescribed distance from a given color in each TIFF image. The particular values used were (100,100,120) color in the RGB space with tolerance of 16% of maximum possible intensity for *hbae1* and 10% for *c-myb*. Calculations were carried out by ImageMagick raster image processing software suite controlled by a Bash script that automated processing and collecting of data. Images were cropped prior to the procedure in order to include the whole CHT region and to prevent accidental misclassification of pixels without signal. Masks consisting of classified pixels were visually inspected for accuracy.

In order to quantify the level of *tp53* WISH signal in the CHT at 72 hpf, two observers independently classified the larvae into four groups according to the strength of the signal in a blinded manner. The difference between the signal in *nol9* ^*sa1022/sa1022*^ larvae and wt siblings, assessed with Mann-Whitney U test, was significant (p<0.05) in each case.

### Whole-mount immunohistochemistry

Whole-mount immunohistochemistry was performed as described before [86], with incubation in 0.1% collagenase (Sigma) in PBS-Tween for 30 min for the digestion step. For immunohistochemistry against glucagon and somatostatin, larvae were deyolked before incubation in blocking buffer. Primary antibodies used were anti-cytokeratin (Santa Cruz Biotechnology, 1:50), anti-glucagon (Sigma, 1:1,000), anti-somatostatin (Dako, 1:200) and anti-carboxypeptidase-a (Sigma, 1:100). The larvae were mounted with Vectashield Mounting Media (Vector Laboratories).

### Microscopy

Images of live larvae, as well as larvae treated with WISH and histochemical stains, were taken using either a Leica DFC 450 CCD camera attached to a Leica LM80 or MZ16 FA dissecting microscope and Leica Application Suite software (Leica Microsystems, Germany), or an AxioCam ICc1 camera attached to a Leica AxioZoom.V16 microscope with ZEN software (Carl Zeiss, Germany).

Confocal images were captured using a Leica TCS SP5 confocal microscope with Leica LAS AF software (Leica Microsystems), using a 20x or 40x lens. The volume of the *ptf1a*-positive exocrine pancreas in *Tg(ptf1a*:*EGFP)* larvae was measured from a z-stack of confocal pictures, using ImageJ 1.48v programme, with a 10 μm slice interval using the Measure Stacks plugin in the ImageJ64 software (National Institutes of Health (NIH), http://imagej.nih.gov/ij/).

### 5-bromo-2'-deoxyuridine (BrdU) incorporation assay

Embryos at either 48- or 72 hpf were incubated in 10mM BrdU (Sigma Aldrich) for 20 min on ice, washed in egg water and incubated at 28°C for 3 h (48 hpf) or 5h (72 hpf). The embryos were then used for immunohistochemistry with anti-GFP primary antibody (Invitrogen, 1:200) and anti-rabbit secondary antibody conjugated to Alexa Fluor 488 (Invitrogen, 1:200), followed by fixation with 4% paraformaldehyde at room temperature for 15 min. The embryos were then cut and their heads were used for genotyping. Embryos of the same genotype were pooled together and subjected to washes with water followed by treatment with 2N HCl for 1h at room temperature. The embryos were then washed consecutively with PBS-Tween and 1M Tris-HCl pH 9.5 and subjected to immunohistochemistry against BrdU with rat anti-BrdU antibody (Abcam, 1:200) and anti-rat antibody conjugated to Cy3 (Millipore, 1:400). Fluorescence was visualized using confocal microscopy.

### TUNEL assay

For assessment of apoptosis in the pancreas, the TUNEL assay was performed on 96 hpf larvae using *In situ* Cell Death Detection Kit, TMR Red (Roche). Fixed larvae were digested in 0.1% collagenase (Sigma) before staining. The larvae were counterstained with DAPI and imaged using confocal microscopy.

For assessment of apoptosis in the CHT of 96 hpf larvae, *In situ* Cell Death Detection Kit, AP (Roche) was used. After incubation with TUNEL reaction mixture, larvae were heated to 80°C for 10 min. After blocking in a solution of 5% heat inactivated Fetal Bovine Serum in PBS-Tween, the larvae were incubated overnight with anti-fluorescein Fab fragments conjugated to alkaline phosphatase (Roche, 1:2,000) and stained with NBT/BCIP solution (Roche), according to the manufacturer’s protocol.

### Flow cytometry

The progeny from a *Tg(c-myb*:*EGFP/nol9* ^*+/sa1022*^ x *nol9* ^*+/sa1022*^ cross at 96 hpf was screened for the presence of EGFP and subjected to immunohistochemical staining with anti-GFP primary antibody (Invitrogen, 1:200) and anti-rabbit secondary antibody conjugated with Alexa Fluor 488 (Invitrogen, 1:200). Afterwards, each larva was dissected: the CHT was stored and the rest of the body was used for genotyping for the *nol9* ^*sa1022*^ allele. CHTs from 8 larvae of the same genotype were pooled, incubated for 30 min with 10 mM DTT (Life Technologies) in Danieau’s solution, followed by incubation with 50 μg/ml liberase (Roche) in PBS for 3 h at 37°C. Single-cell suspension was prepared by passing the solution through a 40 μm mesh cell strainer (Becton Dickinson). Flow cytometry was performed on a BD LSR-Fortessa analyzer (Becton Dickinson). Non-transgenic siblings subjected to immunohistochemical staining were used as a negative control.

### Western blot analysis

Western blotting was performed using the NuPAGE SDS-PAGE gel system (Novex), according to the manufacturer’s protocol. The following antibodies were used at the dilutions indicated: anti-phospho-4EBP1 (Cell Signalling, 1:200) and anti-β-actin (Sigma Aldrich, 1:1,000). Secondary antibodies conjugated with horseradish peroxidase (HRP) were used at 1:50,000 dilution. The signal was developed with a SuperSignal West Femto Substrate kit (Thermo Scienctific) and visualized using an ImageQuant LAS 4000 instrument (GE Healthcare Life Sciences).

### Transmission electron microscopy (TEM)

For TEM, 120 hpf larvae from a cross of *Tg(ptf1a*:*EGFP);nol9* ^*+/sa1022*^ fish were genotyped and fixed in 2% glutaraldehyde and 2% formaldehyde in 0.05 M cacodylate buffer at pH 7.4 for 6 hours at 4°C. They were then processed for infiltration with Quetol epoxy resin as described previously [87]. Images were taken on an FEI Tecnai G2 operated at 120Kv using an AMT XR60B digital camera running Deben software. Transverse sections through the caudal hematopoietic tissue (CHT), as well as longitudinal sections through the pancreas were obtained.

### Histochemical stains

Alcian blue staining was performed on 120 hpf larvae as described previously [88].

*o*-Dianisidine staining for globin was performed as previously described [89]. The *o*-Dianisidine signal was quantified using an analogous method to the one used for the quantification of *c-myb* and *hbae1* WISH signal.

Sudan Black B staining for visualization of neutrophil granules was performed as previously described [90].

### Chemical treatment

*N*-[*N*-(3,5-Difluorophenacetyl)-_L_-alanyl]-*S*-phenylglycine *t*- butyl ester (DAPT, StressMarq) was used to inhibit Notch signaling. Larvae were incubated in 100 μM DAPT in egg water containing PTU from 72 hpf until 120 hpf.

For studies involving the mTor pathway, embryos from a cross of *nol9* ^*+/sa1022*^ fish were incubated in 100 mM solution of L-Leucine, D-Leucine or L-Alanine (Sigma) in egg water with addition of PTU from 24 hpf until 120 hpf.

### Northern blot

Total RNA from phenotypic and non-phenotypic larvae at 120 hpf was isolated using phenol:chloroform extraction. 2 μg of total RNA per lane was subjected to electrophoresis on a 0.8% agarose gel (with the addition of 7% formaldehyde) in 1xMOPS/1% formaldehyde buffer. RNA was then blotted to a Hybond-Nylon membrane (Amersham) and subsequently visualized using methylene blue staining. The detection of rRNA precursors was performed using the High Prime DNA Labeling and Detection Starter Kit II (Roche) kit, according to manufacturer’s protocol. The DNA probes targeted 5’ETS, ITS1, and ITS2 rRNA [61]. The signal was detected using an X-ray film (Kodak).

### Analysis of 28S and 18S rRNA levels

Total RNA extracted from 120 hpf *nol9* ^*sa1022/sa1022*^ larvae and their wt siblings was analyzed on an Agilent Bioanalyser 2100 according to the manufacturer’s instructions.

### Analysis of protein synthesis

Global protein synthesis was assessed using puromycin incorporation [91]. An equal number of *nol9* ^*sa1022/sa1022*^ and wt 120 hpf larvae were suspended in blocking solution (5% FBS / PBS), with the addition of 20 μg/ml puromycin (Sigma) and 100 μg/ml cycloheximide (Santa Cruz Biotechnology), where applicable. Larvae were dissociated by passing through 100 μm mesh and the cell suspension was incubated at room temperature for 10 min, followed by centrifugation at 2,000 rpm for 3 min. The pellets were re-suspended in blocking solution (with the addition of 100 μg/ml cycloheximide, where applicable) and incubated at 28°C for 40 min. After centrifugation, cell pellets were used for Western blot analysis using anti-puromycin antibody (1:150, kind gift from Yusuke Sekine) and anti-β-actin antibody (Sigma Aldrich, 1:1,000) for loading control. Signal strength was quantified by densitometry measurements using ImageJ. For β-actin, the single band was quantified. For puromycin, an area of gel with the weakest background (the signal in the no puromycin control) was chosen. For each lane, the full signal within the chosen area was measured and the value for the background was subtracted. The puromycin signal value was then divided by the β-actin value for the same sample.

### Gene knock-down with morpholinos (MOs)

Morpholinos (Gene-Tools) were diluted in distilled water with 0.25% phenol red (Sigma) and injected into 1- to 2-cell stage embryos at 4 ng. The MO sequences were: *nol9* ATG MO: 5’- ACCTTGTGTACTTTCATTGTCATCC-3’, Std Ctrl MO 5’-CCTCTTACCTCAGTTACAATTTATA-3’.

### Statistical methods

Statistical analyses were performed in Microsoft Excel and Statplus (AnalystSoft).

## Supporting Information

S1 Fig *nol9* mutation affects the development of liver and intestine but not of jaw cartilage. (A) Representative pictures of 96 hpf larvae stained by WISH against the liver marker *prox1* (black arrow). Magnified images of the liver are shown. (B) Quantification of the *prox1 in situ* hybridization data. The average *prox1*-positive area is decreased in *nol9* ^*sa1022/sa1022*^ mutants (n = 13) compared to wild-type siblings (n = 25). Data are represented as the average +/- SEM. Two-tailed Student’s *t* test, ***, p<0.001. (C) Representative images of 96 hpf larvae stained by WISH against the intestinal marker *fabp2a*. The expression of *fabp2a* (blue arrow) is decreased in *nol9* ^*sa1022/sa1022*^ (n = 11) compared to wild-type siblings (n = 32). (D) Alcian blue staining, showing normal formation of the jaw cartilage elements Meckel’s (m), palatoquadrate (pq), ceratohyal (ch) and ceratobranchial (cb) in both *nol9* ^*sa1022/sa1022*^ mutants (n = 23) and wt siblings (n = 54) at 120 hpf. Ventral view with anterior to the left.
(TIFF)

S2 Fig Definitive, but not primitive erythropoiesis is affected in *nol9* ^*sa1022/sa1022*^ mutants. (A-D) *o*-Dianisidine staining of circulating erythrocytes at 48 hpf, 96 hpf and 120 hpf. (A) Representative images of *o*-Dianisidine-stained embryos at 48 hpf. The level of staining was similar between *nol9* ^*+/+*^ (n = 13), *nol9* ^*+/sa1022*^ (n = 35) and *nol9* ^*sa1022/sa1022*^ (n = 8) embryos. Ventral view with anterior up. (B) Representative images of *o*-Dianisidine-stained larvae at 96 hpf. The number of stained circulating primitive erythrocytes was comparable between *nol9* ^*sa1022/sa1022*^ (n = 10), *nol9* ^*+/+*^ (n = 15) and *nol9* ^*+/sa1022*^ (n = 15) siblings. Ventral view with anterior up. (C) Quantification of the area stained in *o*-Dianisidine-stained larvae area at 96 hpf. Data are represented as average +/- SEM. Two-tailed Student’s *t* test, p>0.05. ns–not significant. (D) Representative images of *o*-Dianisidine-stained larvae at 120 hpf. All *nol9* ^*sa1022/sa1022*^ (n = 20), *nol9* ^*+/+*^ (n = 2) and *nol9* ^*+/sa1022*^ (n = 8) larvae displayed a similar level of staining. Larvae oriented with anterior to the left and dorsal to the top. (E) Whole-mount *in situ* hybridization using *hbae1* riboprobe at 96 hpf. Arrows indicate *hbae1*-positive definitive erythrocytes present in the CHT of *nol9* ^*+/sa1022*^ and *nol9* ^*+/+*^ but not in *nol9* ^*sa1022/sa1022*^ larvae. (F) Quantification of *hbae1 in situ* hybridization data. Data are represented as the number of larvae belonging to each phenotypic group. Fisher’s exact test, **, p<0.01.
(TIFF)

S3 Fig *nol9* mutation affects the 28S/18S rRNA ratio. (A) Representative E-Bioanalyser analysis of total RNA isolated from *nol9* ^*sa1022/sa1022*^ mutants and their wt siblings at 120 hpf. Peaks corresponding to 18S (arrowhead) and 28S (arrow) are indicated. (B) 28S/18S ratio in *nol9* ^*sa1022/sa1022*^ larvae and wt siblings, based on the E-Bioanalyser analysis of total RNA. Data are represented as average +/- SD, n_replicates_ = 4, paired Student’s *t*-test, *, p<0.05.
(TIFF)

S4 Fig *nol9* ^*sa1022/sa1022*^ mutants have a defect in development of the exocrine but not the endocrine pancreas. (A) Representative confocal images of the pancreas of *Tg(ptf1a*:*EGFP;ins*:*mCherry)* fish at 48-, 72- and 120 hpf. No difference was observed between *nol9* ^*sa1022/sa1022*^ and wt siblings at 48- and 72 hpf. At 120 hpf the area of the *ptf1a* ^*+*^ exocrine pancreas (green) was decreased in *nol9* ^*sa1022/sa1022*^ larvae, while the area of *ins* ^*+*^ endocrine pancreas (red) was comparable in *nol9* ^*sa1022/sa1022*^ mutants and wt siblings. (B) Confocal images of the endocrine pancreas of larvae subjected to immunohistochemistry against Somatostatin and Glucagon at 96 hpf. The intensity and area covered by the signal was comparable in *nol9* ^*sa1022/sa1022*^ larvae and wt siblings for both antibodies. (C) Average volume of the *ptf1a*-positive exocrine pancreas in *nol9* ^*sa1022/sa1022*^ (n = 8) and wt (n = 15) larvae at 120 hpf. Data are represented as the mean +/- SEM, Student’s *t*-test, **, p<0.01. All images are oriented with anterior to the right and dorsal to the top.
(TIFF)

S5 Fig Gene knockdown studies using *nol9* morpholino confirm the pancreas phenotype observed in *nol9* ^*sa1022/sa1022*^ mutants. (A) General morphology of 96 hpf larvae injected with either standard control morpholino (std MO) or a morpholino targeting the translational start site of the *nol9* transcript (*nol9* ATG MO). Numbers represent larvae with the displayed phenotype out of the total number of larvae examined. (B) Representative confocal images of the pancreas of 96 hpf *Tg(ptf1a*:*EGFP;ins*:*mCherry)* larvae injected with std MO or *nol9* ATG MO. While the *ptf1a* ^+^ exocrine pancreas appeared smaller in larvae injected with *nol9* ATG MO compared to std MO, the *ins* ^+^ endocrine pancreas was comparable between the groups. (C) The average volume of the *ptf1a* ^+^ exocrine pancreas in 96 hpf larvae injected with std MO (n = 19) or *nol9* ATG MO (n = 25). Data are represented as the mean +/- SEM, Student’s *t*-test, **, p<0.01.
(TIFF)

S6 Fig Global protein synthesis in zebrafish larvae assessed by western blotting for puromycin incorporation. (A) Western blot analysis of puromycin following its incorporation into newly synthesized proteins in *nol9* ^*sa1022/sa1022*^ mutants and wt siblings at 120 hpf. β-actin was detected as a loading control. P–puromycin, CHX–cycloheximide. (B) The average intensity of puromycin signal, standardized by β-actin signal. Data are represented as the mean +/- SEM. Student’s *t*-test, **, p<0.01, ns–not significant, CHX—cycloheximide.
(TIFF)

S7 Fig Formation of secondary islets and apoptosis levels in pancreas are unaffected in *nol9* ^*sa1022/sa1022*^ mutants. (A) Representative confocal images of the pancreas of 120 hpf *Tg(ptf1a*:*EGFP;ins*:*mCherry)* larvae showing the presence of *ins*-expressing secondary islets (arrow) in *nol9* ^*sa1022/sa1022*^ mutants and wt siblings. (B) The percentage of *Tg(ptf1a*:*EGFP;ins*:*mCherry)* larvae whose pancreas contained secondary islets, depending on their genotype and previous treatment with either DAPT inhibitor or DMSO (vehicle control) at 120 hpf. The total number of larvae in each group is indicated. (C) Representative confocal images of *Tg(ptf1a*:*EGFP)* larvae subjected to TUNEL assay at 96 hpf and co-stained with DAPI. No TMR-labelled apoptotic cells were observed in the *ptf1a*-expressing exocrine pancreas of *nol9* ^*sa1022/sa1022*^ mutants (n = 8) or their wt siblings (n = 9). However, the tails of *nol9* ^*sa1022/sa1022*^ mutants and wt siblings contained similar numbers of apoptotic cells (arrow). (D) The mean number of apoptotic cells in the tails of *nol9* ^*sa1022/sa1022*^ mutants (n = 8) and wt siblings (n = 9). Data are represented as the mean +/- SEM; Student’s *t*-test, ns–not significant.
(TIFF)

S8 Fig Proliferation of exocrine pancreas cells in *nol9* ^*sa1022/sa1022*^ mutants. (A) Representative confocal images of *Tg(ptf1a*:*EGFP)* larvae subjected to BrdU incorporation assay at 96 hpf. Double positive *ptf1a* ^*+*^ BrdU^+^ cells are indicated with arrows. Images are oriented with anterior to the right and dorsal to the top. (B) Average number of *ptf1a* ^+^ cells which incorporated BrdU, normalized to the volume of *ptf1*a^+^ exocrine pancreas, in *nol9* ^*sa1022/sa1022*^ (n = 9) and wt (n = 7) larvae at 96 hpf. Data are represented as the mean +/- SEM. Student’s *t*-test, p = 0.051.
(TIFF)

S9 Fig The number of thrombocytes and neutrophils is unaffected in *nol9* ^*sa1022/sa1022*^ mutants. (A) Representative maximum projection confocal images showing thrombocytes (GFP^bright^, arrow) and HSPCs (GFP^dim^, arrowhead) in the CHT of *Tg(cd41*:*EGFP)* larvae at 96 hpf. Larvae are oriented with anterior to the left and dorsal to the top. (B) The number of GFP^bright^ thrombocytes observed in the CHT region of *nol9* ^*sa1022/sa1022*^ mutants (n = 20) and their wt siblings (n = 51) at 96 hpf. Data are represented as the mean +/- SEM; Student’s *t*-test, p>0.05. (C) Images of the CHT region of 72 hpf larvae stained with Sudan Black B. Stained neutrophils are marked by an arrow. (D) The average number of Sudan Black B-stained neutrophils in the CHT region of 72 hpf larvae, depending on their genotype (*nol9* ^*+/+*^ n = 32, *nol9* ^*+/sa1022*^ n = 32 and *nol9* ^*sa1022/sa1022*^ n = 28). Data are represented as the mean +/- SEM; One-way ANOVA, ns–not significant.
(TIFF)

S10 Fig Analysis of the number of *c-myb*+ cells in the CHT of *nol9* ^*sa1022/sa1022*^ mutants. (A) Representative results of flow cytometric analysis of the number of *c-myb* ^*+*^ cells in the CHT of *nol9* ^*sa1022/sa1022*^ and *nol9* ^*+/+*^ larvae, using the progeny of a *nol9* ^*+/sa1022*^ *Tg(c-myb*:*EGFP)* x *nol9* ^*+/sa1022*^ cross at 96 hpf. Non-transgenic siblings were included as a negative control. Gating of the GFP^+^ population is shown. (B) The number of *c-myb* ^*+*^ cells in the CHT of 96 hpf *Tg(c-myb*:*EGFP) nol9* ^*sa1022/sa1022*^ and *nol9* ^*+/+*^ larvae analysed by flow cytometry. Data are represented as the percentage of GFP^+^ cells within the single cell population. Student’s *t*-Test, n = 2, *, p<0.05. (C) Average intensity of the GFP signal for *c-myb* ^*+*^ cells in the CHT of 96 hpf *Tg(c-myb*:*EGFP) nol9* ^*sa1022/sa1022*^ and *nol9* ^*+/+*^. Only the cells gated as shown in (A) were included. Data represented as average +/- SEM. Student’s *t*-Test, n = 2, p>0.05, ns–not significant.
(TIFF)

S11 Fig Apoptosis in the CHT assessed by TUNEL assay. (A) The CHT region of 72 hpf larvae stained with TUNEL assay. *nol9* ^*sa1022/sa1022*^ mutants do not exhibit an increase in the number of TUNEL-positive apoptotic cells (arrow) compared to wt siblings in the CHT region (as outlined). All larvae are oriented with anterior to the left and dorsal to the top. (B) The average number of TUNEL-positive apoptotic cells in the CHT region in *nol9* ^*+/+*^ (n = 16), *nol9* ^*+/sa1022*^ (n = 34) and *nol9* ^*sa1022/sa1022*^ (n = 15) larvae at 72 hpf. Data are represented as the mean +/- SEM; One way ANOVA, ns–not significant.
(TIFF)

S12 Fig The pancreatic and hematological defects in *nol9* ^*sa1022/sa1022*^ larvae are independent of the mTOR pathway. (A) Western blot analysis of p4EBP1 and β-actin (loading control) in whole cell lysates of *nol9* ^*sa1022/sa1022*^ and wt siblings at 120 hpf. (B) Representative confocal images of the pancreas of *Tg(ptf1a*:*EGFP) nol9* ^*sa1022/sa1022*^ and *nol9* ^*+/+*^ larvae at 120 hpf after treatment with L-Leucine or D-Leucine from 24 hpf. (C) The average volume of the *ptf1a*-positive exocrine pancreas in 120 hpf *Tg(ptf1a*:*EGFP)* larvae, depending on their genotype and treatment with either D-Leucine or L-Leucine from 24 hpf. The data are represented as the mean +/- SEM. Student’s *t*-test, **, p<0.01. (D) Quantification of *hbae1* WISH performed on 120 hpf larvae treated with D-Leucine, L-Leucine or L-Alanine from 24 hpf. Data are represented as the number of wt (*nol9* ^*+/+*^/*nol9* ^*+/sa1022*^) or mutant (*nol9* ^*sa1022/sa1022*^) larvae belonging to either phenotypic group. Fisher’s exact test, **, p<0.01.
(TIFF)

S13 Fig The exocrine pancreas phenotype of *nol9* ^*sa1022/sa1022*^ mutants is independent of Tp53 signalling. (A) Confocal images of the pancreas of 120 hpf larvae from a *Tg(ptf1a*:*EGFP)*;*nol9* ^*+/sa1022*^ *;tp53* ^*+/zdf1*^ x *nol9* ^*+/sa1022*^ *;tp53* ^*+/zdf1*^ cross. (B) The average volume of the *ptf1a* ^+^ exocrine pancreas of 120 hpf larvae from a *Tg(ptf1a*:*EGFP)*;*nol9* ^*+/sa1022*^ *;tp53* ^*+/zdf1*^ x *nol9* ^*+/sa1022*^ *;tp53* ^*+/zdf1*^ cross, depending on their genotype. Data are represented as the mean +/- SEM. Student’s *t*-test, **, p<0.01, ns–not significant. Within this figure, *nol9* ^*sa1022*^ allele has been denoted as *nol9* ^*-*^ and *tp53* ^*zdf1*^ as *tp53* ^*-*^.
(TIFF)
