# Supplementary figures and images for: The Ribosome Biogenesis Protein Nol9 Is Essential for Definitive Hematopoiesis and Pancreas Morphogenesis in Zebrafish
Source: PLoS Genet. 2015 Dec 1;11(12):e1005677. doi: 10.1371/journal.pgen.1005677 (PMC4666468; doi:10.1371/journal.pgen.1005677)

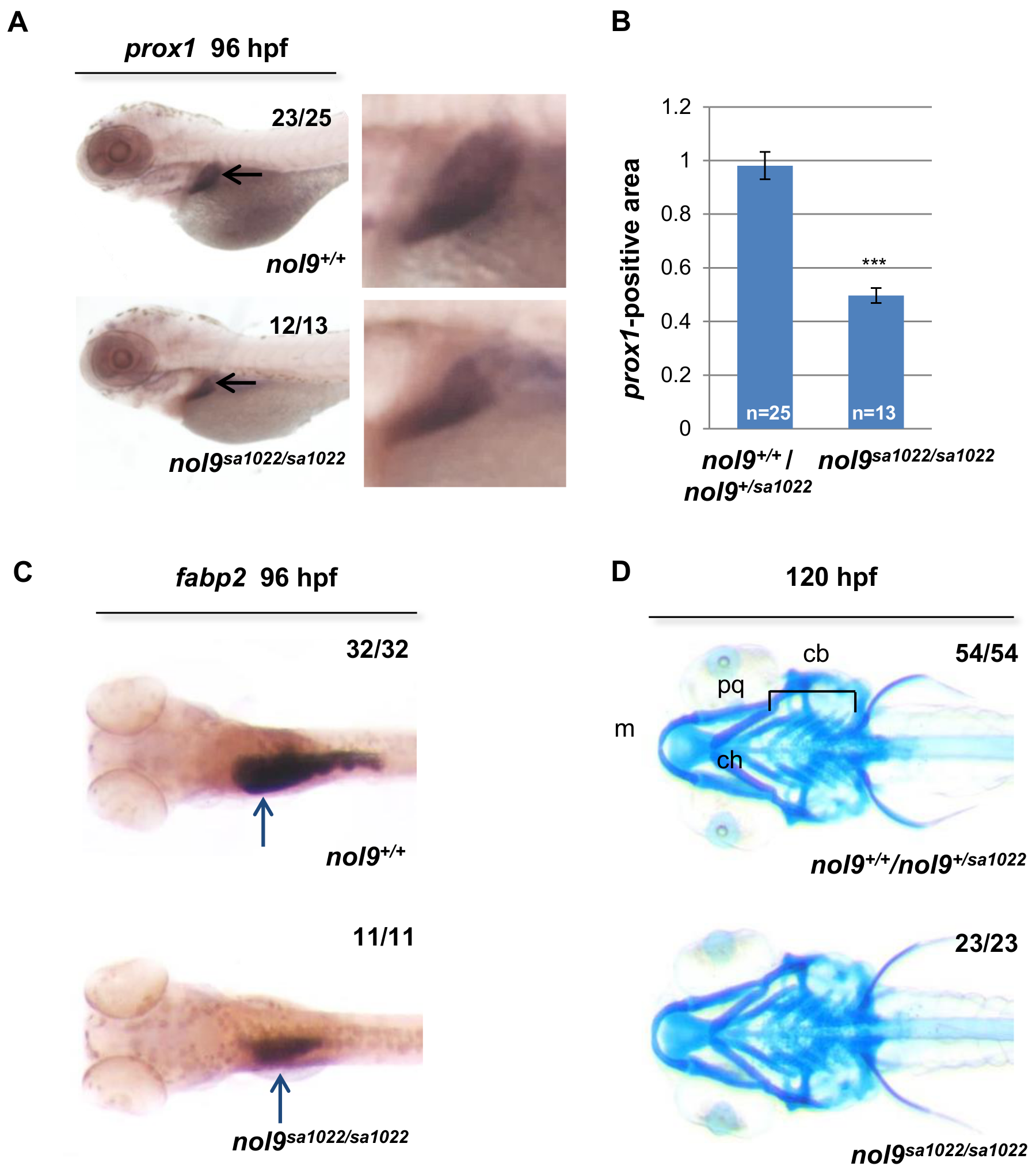

Supplement: S1 Fig — (A) Representative pictures of 96 hpf larvae stained by WISH against the liver marker prox1 (black arrow). Magnified images of the liver are shown. (B) Quantification of the prox1 in situ hybridization data. The average prox1-positive area is decreased in nol9 sa1022/sa1022 mutants (n = 13) compared to wild-type siblings (n = 25). Data are represented as the average +/- SEM. Two-tailed Student’s t test, ***, p<0.001. (C) Representative images of 96 hpf larvae stained by WISH against the intestinal marker fabp2a. The expression of fabp2a (blue arrow) is decreased in nol9 sa1022/sa1022 (n = 11) compared to wild-type siblings (n = 32). (D) Alcian blue staining, showing normal formation of the jaw cartilage elements Meckel’s (m), palatoquadrate (pq), ceratohyal (ch) and ceratobranchial (cb) in both nol9 sa1022/sa1022 mutants (n = 23) and wt siblings (n = 54) at 120 hpf. Ventral view with anterior to the left. (TIFF) [file pgen.1005677.s001.tiff]

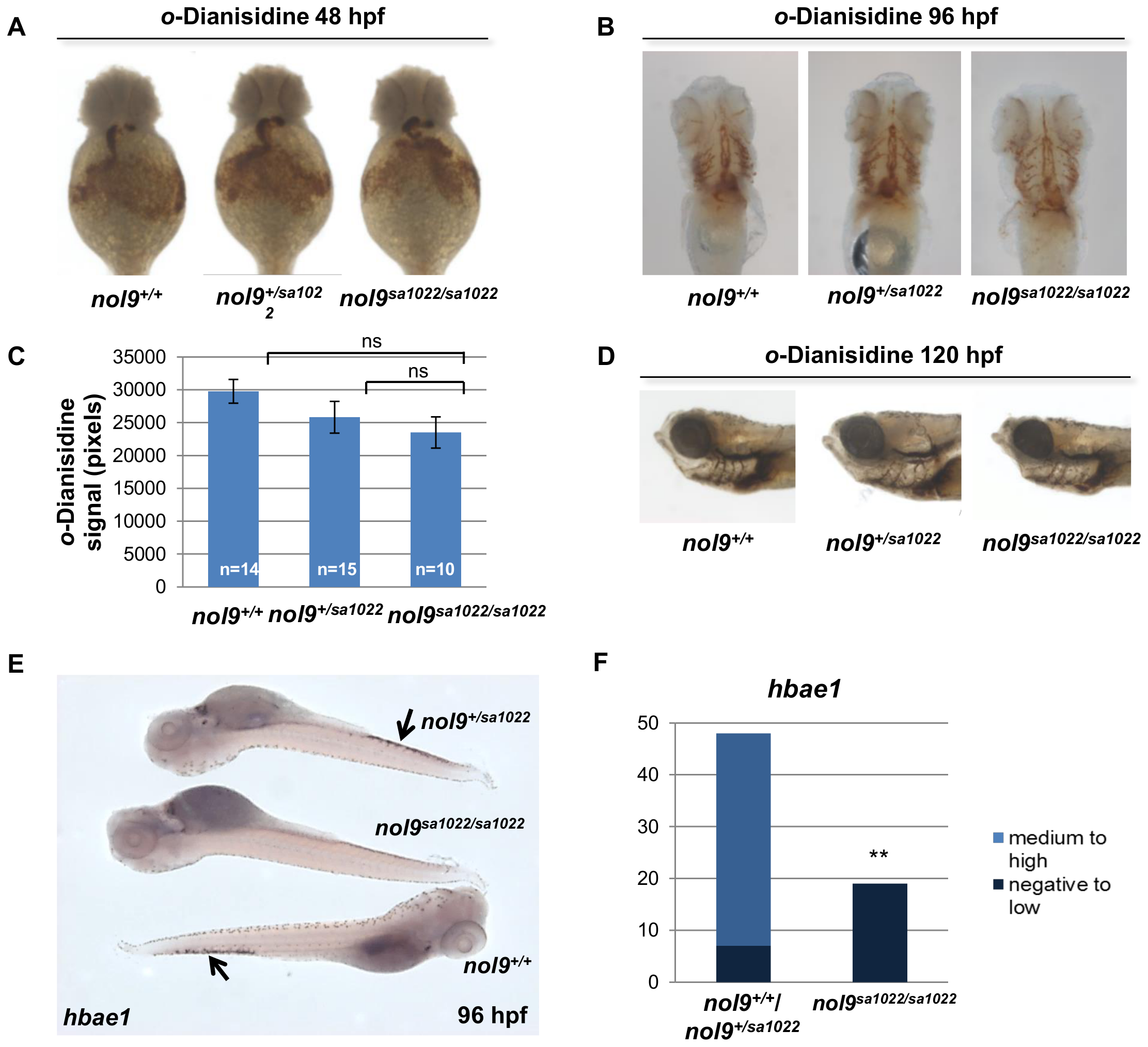

Supplement: S2 Fig — (A-D) o-Dianisidine staining of circulating erythrocytes at 48 hpf, 96 hpf and 120 hpf. (A) Representative images of o-Dianisidine-stained embryos at 48 hpf. The level of staining was similar between nol9 +/+ (n = 13), nol9 +/sa1022 (n = 35) and nol9 sa1022/sa1022 (n = 8) embryos. Ventral view with anterior up. (B) Representative images of o-Dianisidine-stained larvae at 96 hpf. The number of stained circulating primitive erythrocytes was comparable between nol9 sa1022/sa1022 (n = 10), nol9 +/+ (n = 15) and nol9 +/sa1022 (n = 15) siblings. Ventral view with anterior up. (C) Quantification of the area stained in o-Dianisidine-stained larvae area at 96 hpf. Data are represented as average +/- SEM. Two-tailed Student’s t test, p>0.05. ns–not significant. (D) Representative images of o-Dianisidine-stained larvae at 120 hpf. All nol9 sa1022/sa1022 (n = 20), nol9 +/+ (n = 2) and nol9 +/sa1022 (n = 8) larvae displayed a similar level of staining. Larvae oriented with anterior to the left and dorsal to the top. (E) Whole-mount in situ hybridization using hbae1 riboprobe at 96 hpf. Arrows indicate hbae1-positive definitive erythrocytes present in the CHT of nol9 +/sa1022 and nol9 +/+ but not in nol9 sa1022/sa1022 larvae. (F) Quantification of hbae1 in situ hybridization data. Data are represented as the number of larvae belonging to each phenotypic group. Fisher’s exact test, **, p<0.01. (TIFF) [file pgen.1005677.s002.tiff]

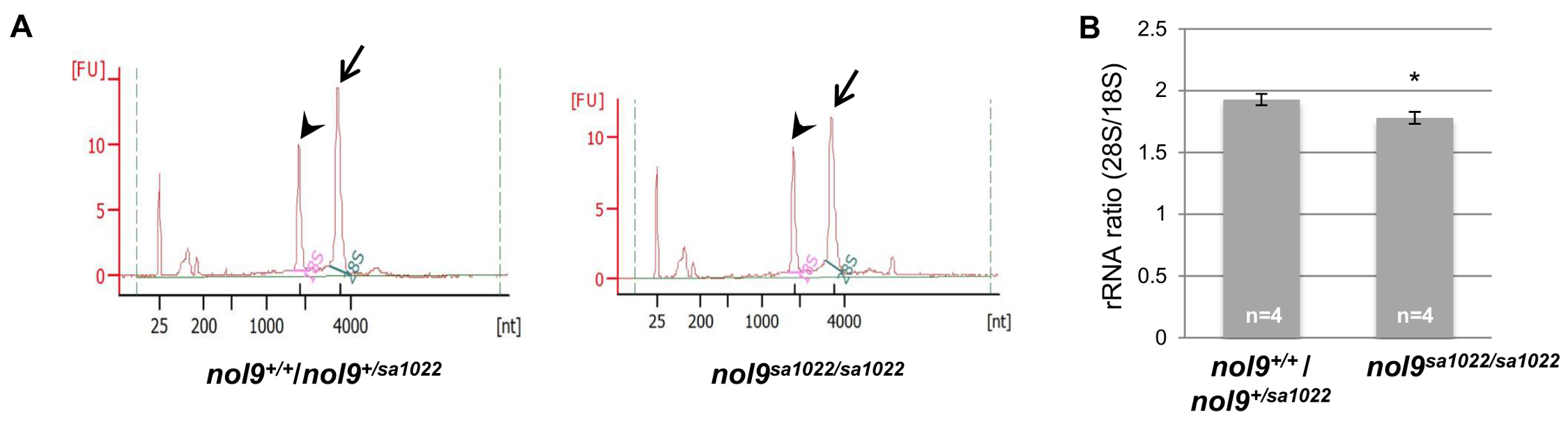

Supplement: S3 Fig — (A) Representative E-Bioanalyser analysis of total RNA isolated from nol9 sa1022/sa1022 mutants and their wt siblings at 120 hpf. Peaks corresponding to 18S (arrowhead) and 28S (arrow) are indicated. (B) 28S/18S ratio in nol9 sa1022/sa1022 larvae and wt siblings, based on the E-Bioanalyser analysis of total RNA. Data are represented as average +/- SD, nreplicates = 4, paired Student’s t-test, *, p<0.05. (TIFF) [file pgen.1005677.s003.tiff]

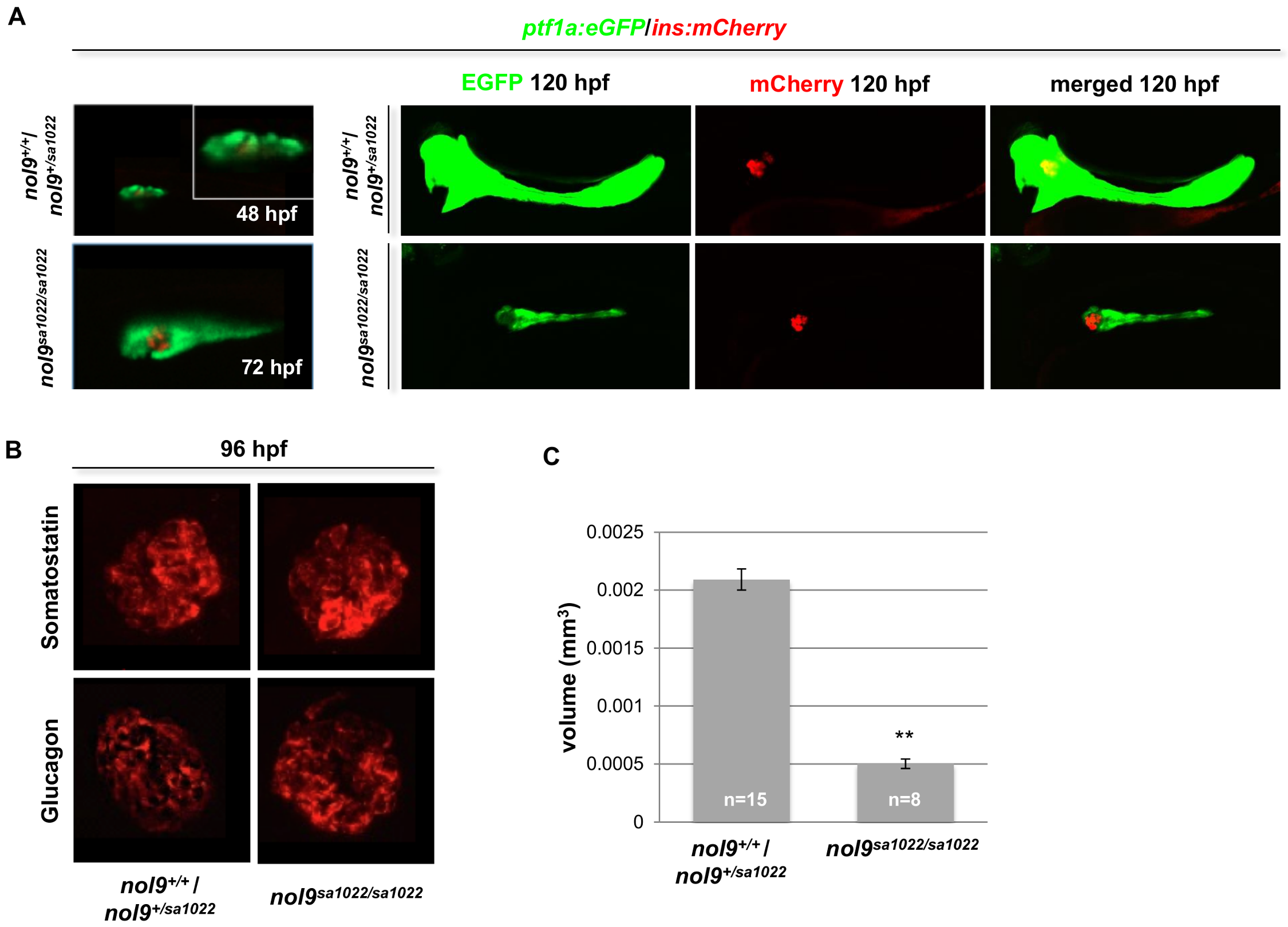

Supplement: S4 Fig — (A) Representative confocal images of the pancreas of Tg(ptf1a:EGFP;ins:mCherry) fish at 48-, 72- and 120 hpf. No difference was observed between nol9 sa1022/sa1022 and wt siblings at 48- and 72 hpf. At 120 hpf the area of the ptf1a + exocrine pancreas (green) was decreased in nol9 sa1022/sa1022 larvae, while the area of ins + endocrine pancreas (red) was comparable in nol9 sa1022/sa1022 mutants and wt siblings. (B) Confocal images of the endocrine pancreas of larvae subjected to immunohistochemistry against Somatostatin and Glucagon at 96 hpf. The intensity and area covered by the signal was comparable in nol9 sa1022/sa1022 larvae and wt siblings for both antibodies. (C) Average volume of the ptf1a-positive exocrine pancreas in nol9 sa1022/sa1022 (n = 8) and wt (n = 15) larvae at 120 hpf. Data are represented as the mean +/- SEM, Student’s t-test, **, p<0.01. All images are oriented with anterior to the right and dorsal to the top. (TIFF) [file pgen.1005677.s004.tiff]

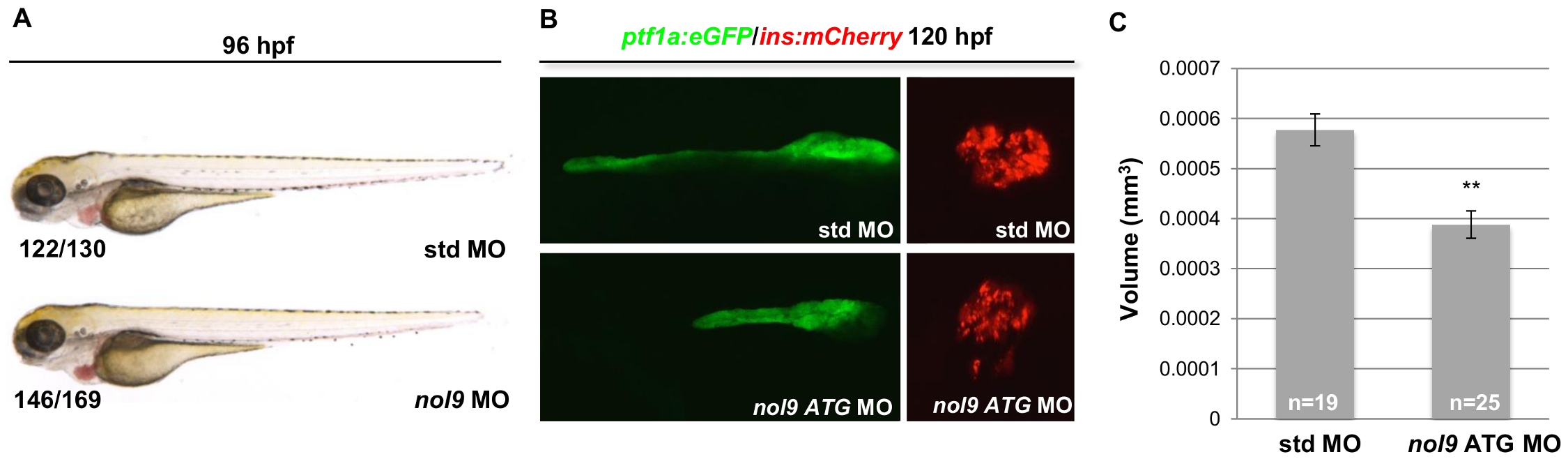

Supplement: S5 Fig — (A) General morphology of 96 hpf larvae injected with either standard control morpholino (std MO) or a morpholino targeting the translational start site of the nol9 transcript (nol9 ATG MO). Numbers represent larvae with the displayed phenotype out of the total number of larvae examined. (B) Representative confocal images of the pancreas of 96 hpf Tg(ptf1a:EGFP;ins:mCherry) larvae injected with std MO or nol9 ATG MO. While the ptf1a + exocrine pancreas appeared smaller in larvae injected with nol9 ATG MO compared to std MO, the ins + endocrine pancreas was comparable between the groups. (C) The average volume of the ptf1a + exocrine pancreas in 96 hpf larvae injected with std MO (n = 19) or nol9 ATG MO (n = 25). Data are represented as the mean +/- SEM, Student’s t-test, **, p<0.01. (TIFF) [file pgen.1005677.s005.tiff]

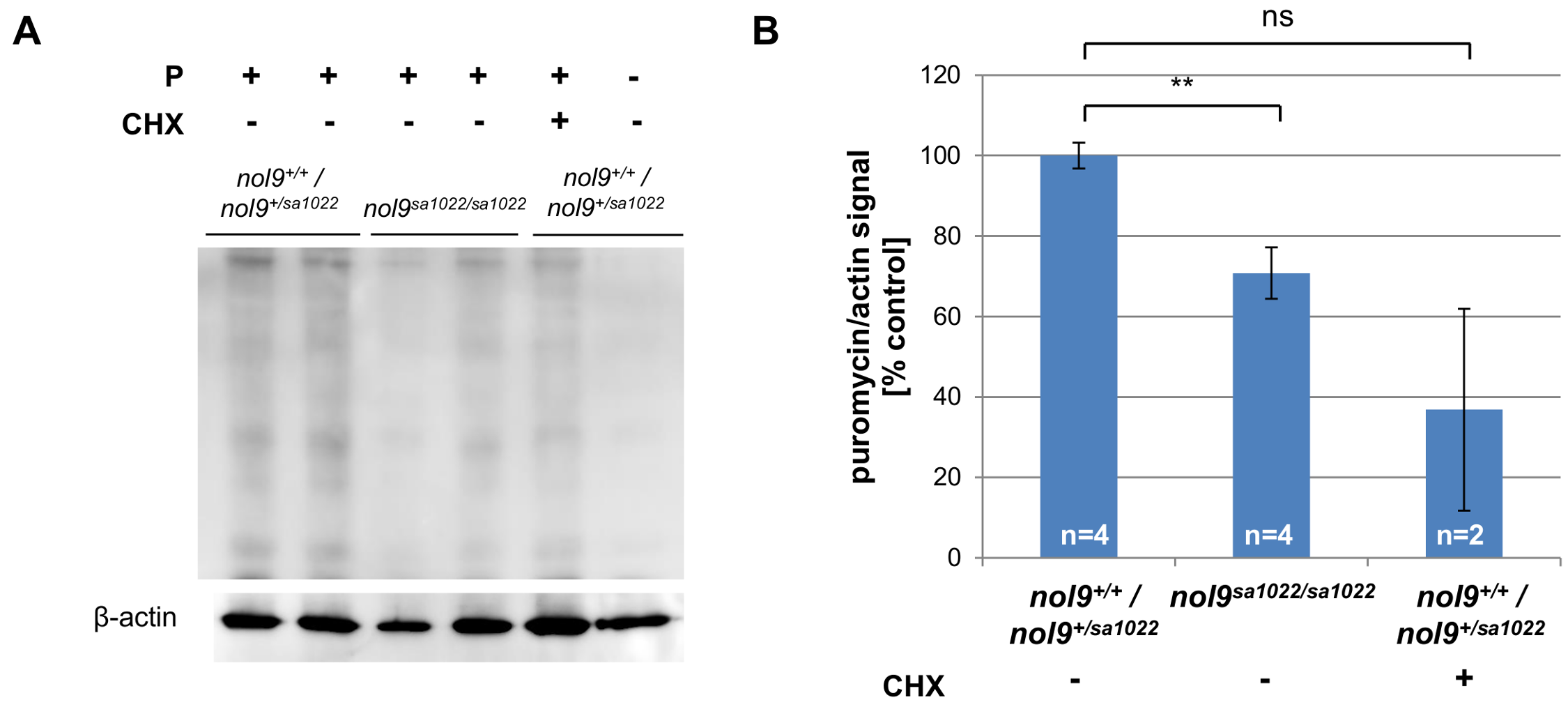

Supplement: S6 Fig — (A) Western blot analysis of puromycin following its incorporation into newly synthesized proteins in nol9 sa1022/sa1022 mutants and wt siblings at 120 hpf. β-actin was detected as a loading control. P–puromycin, CHX–cycloheximide. (B) The average intensity of puromycin signal, standardized by β-actin signal. Data are represented as the mean +/- SEM. Student’s t-test, **, p<0.01, ns–not significant, CHX—cycloheximide. (TIFF) [file pgen.1005677.s006.tiff]

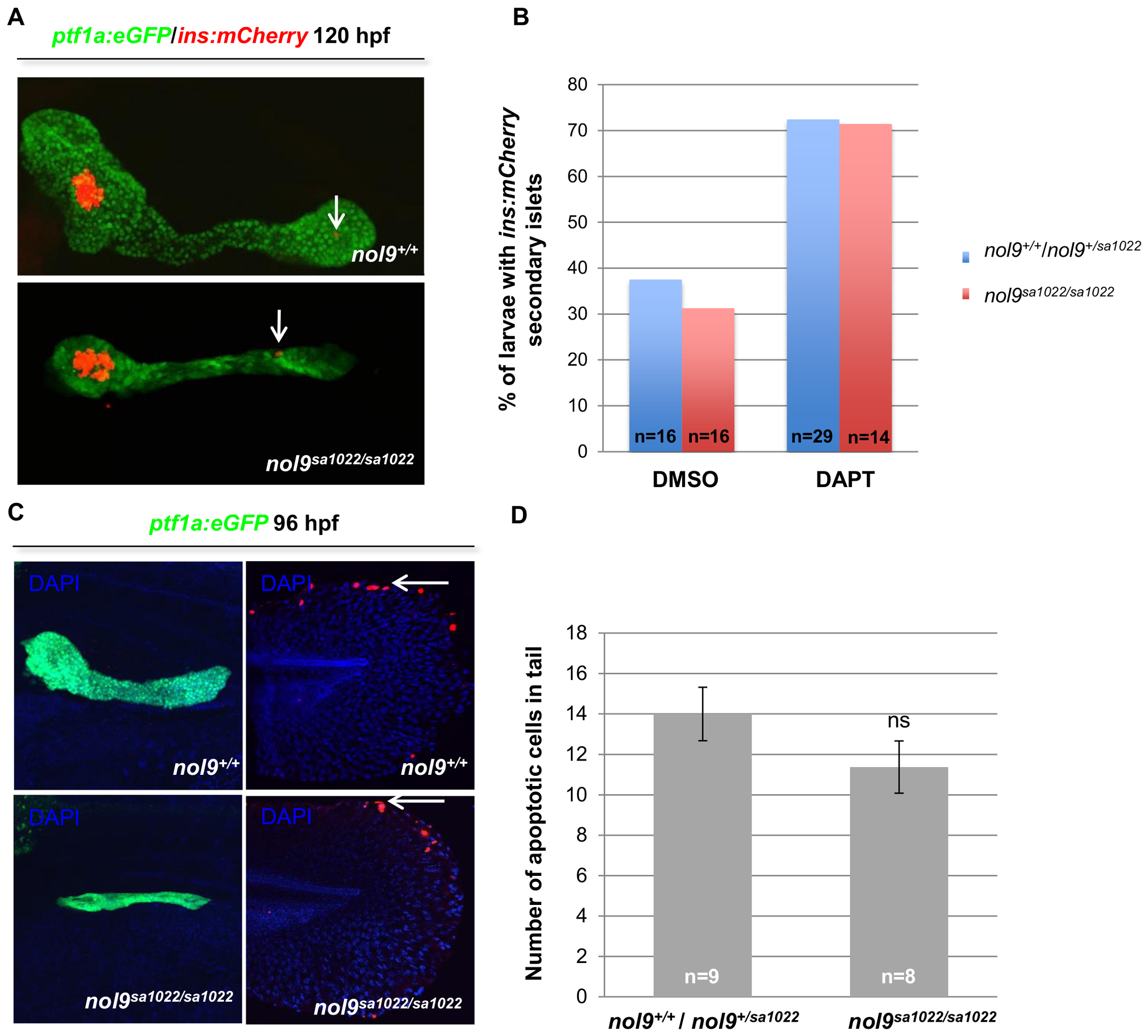

Supplement: S7 Fig — (A) Representative confocal images of the pancreas of 120 hpf Tg(ptf1a:EGFP;ins:mCherry) larvae showing the presence of ins-expressing secondary islets (arrow) in nol9 sa1022/sa1022 mutants and wt siblings. (B) The percentage of Tg(ptf1a:EGFP;ins:mCherry) larvae whose pancreas contained secondary islets, depending on their genotype and previous treatment with either DAPT inhibitor or DMSO (vehicle control) at 120 hpf. The total number of larvae in each group is indicated. (C) Representative confocal images of Tg(ptf1a:EGFP) larvae subjected to TUNEL assay at 96 hpf and co-stained with DAPI. No TMR-labelled apoptotic cells were observed in the ptf1a-expressing exocrine pancreas of nol9 sa1022/sa1022 mutants (n = 8) or their wt siblings (n = 9). However, the tails of nol9 sa1022/sa1022 mutants and wt siblings contained similar numbers of apoptotic cells (arrow). (D) The mean number of apoptotic cells in the tails of nol9 sa1022/sa1022 mutants (n = 8) and wt siblings (n = 9). Data are represented as the mean +/- SEM; Student’s t-test, ns–not significant. (TIFF) [file pgen.1005677.s007.tiff]

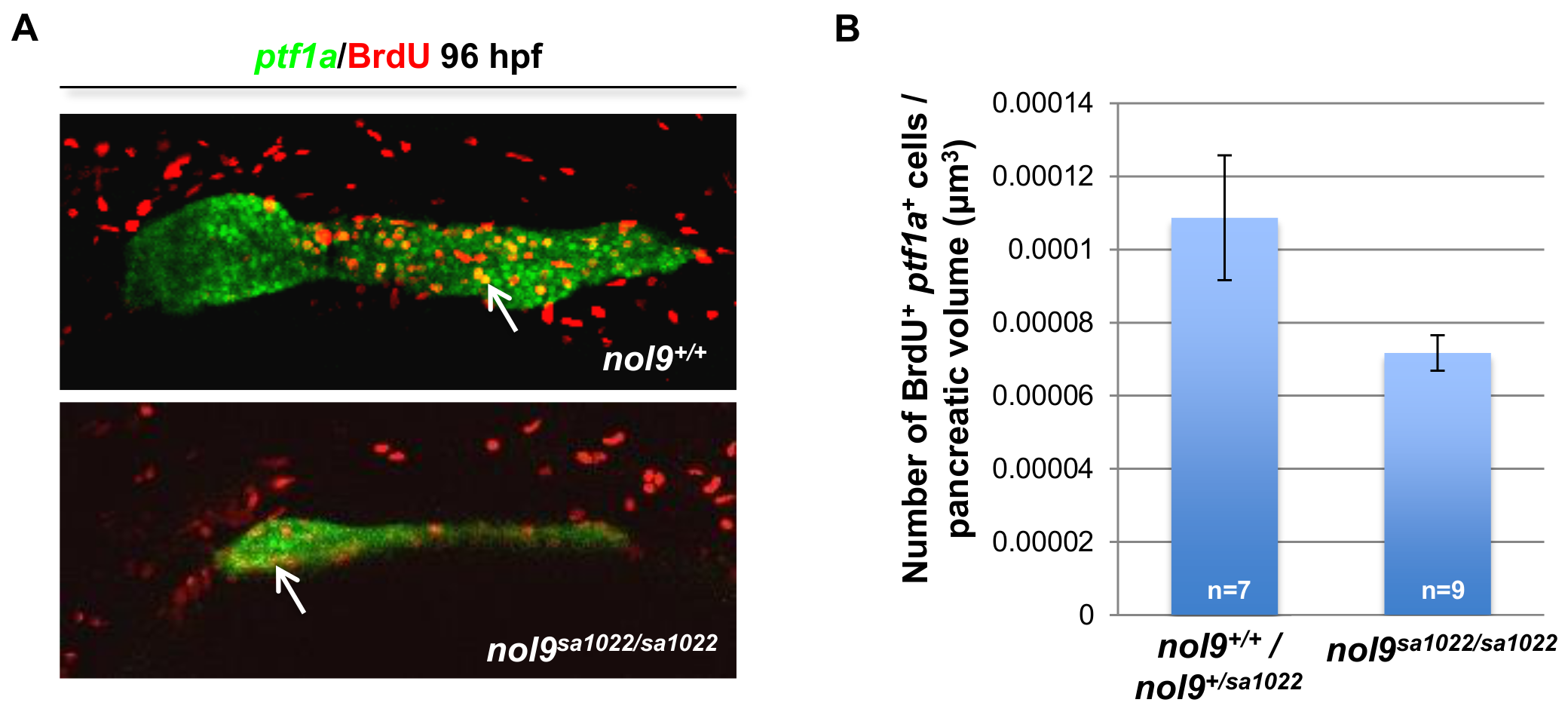

Supplement: S8 Fig — (A) Representative confocal images of Tg(ptf1a:EGFP) larvae subjected to BrdU incorporation assay at 96 hpf. Double positive ptf1a + BrdU+ cells are indicated with arrows. Images are oriented with anterior to the right and dorsal to the top. (B) Average number of ptf1a + cells which incorporated BrdU, normalized to the volume of ptf1a+ exocrine pancreas, in nol9 sa1022/sa1022 (n = 9) and wt (n = 7) larvae at 96 hpf. Data are represented as the mean +/- SEM. Student’s t-test, p = 0.051. (TIFF) [file pgen.1005677.s008.tiff]

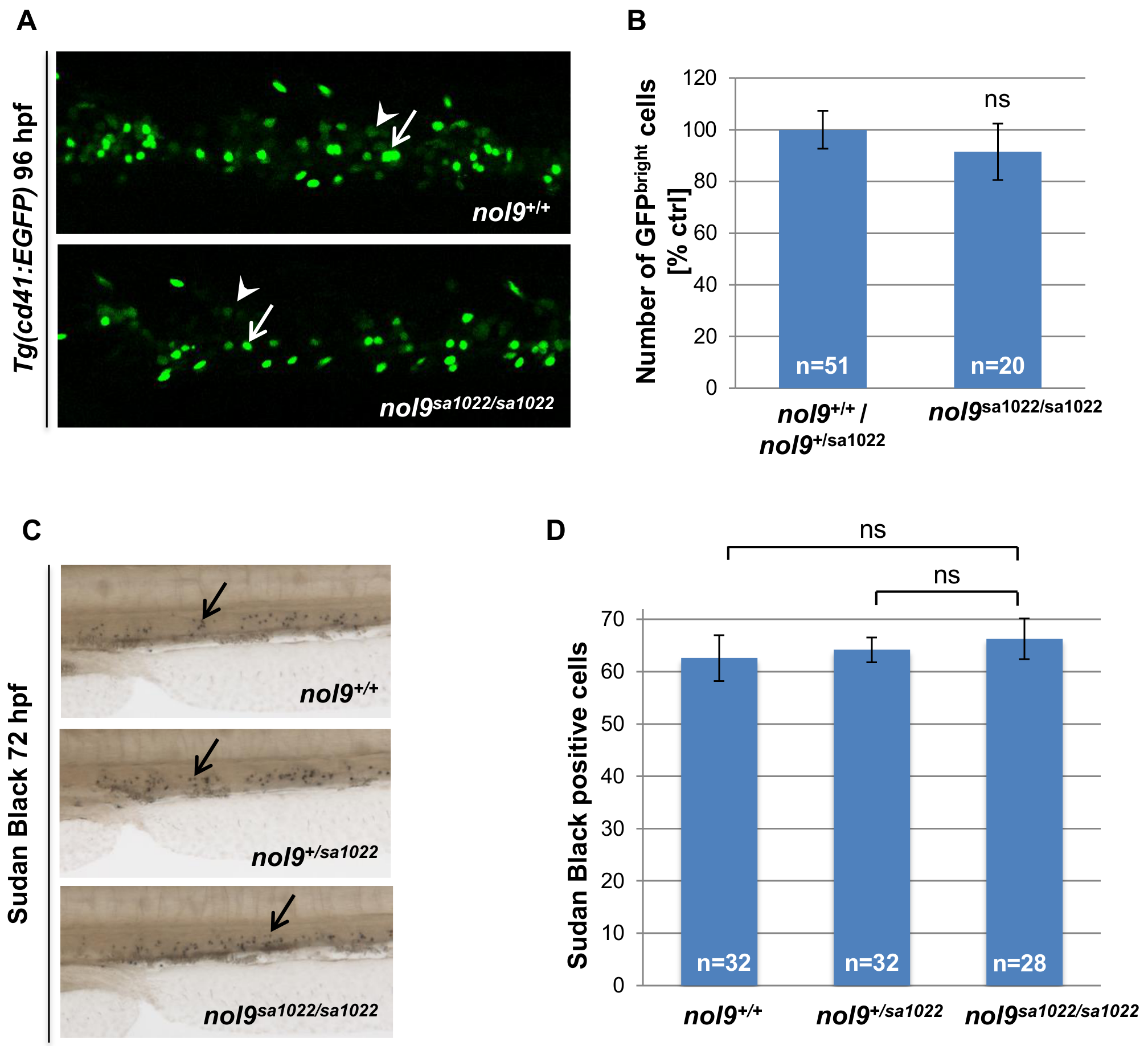

Supplement: S9 Fig — (A) Representative maximum projection confocal images showing thrombocytes (GFPbright, arrow) and HSPCs (GFPdim, arrowhead) in the CHT of Tg(cd41:EGFP) larvae at 96 hpf. Larvae are oriented with anterior to the left and dorsal to the top. (B) The number of GFPbright thrombocytes observed in the CHT region of nol9 sa1022/sa1022 mutants (n = 20) and their wt siblings (n = 51) at 96 hpf. Data are represented as the mean +/- SEM; Student’s t-test, p>0.05. (C) Images of the CHT region of 72 hpf larvae stained with Sudan Black B. Stained neutrophils are marked by an arrow. (D) The average number of Sudan Black B-stained neutrophils in the CHT region of 72 hpf larvae, depending on their genotype (nol9 +/+ n = 32, nol9 +/sa1022 n = 32 and nol9 sa1022/sa1022 n = 28). Data are represented as the mean +/- SEM; One-way ANOVA, ns–not significant. (TIFF) [file pgen.1005677.s009.tiff]

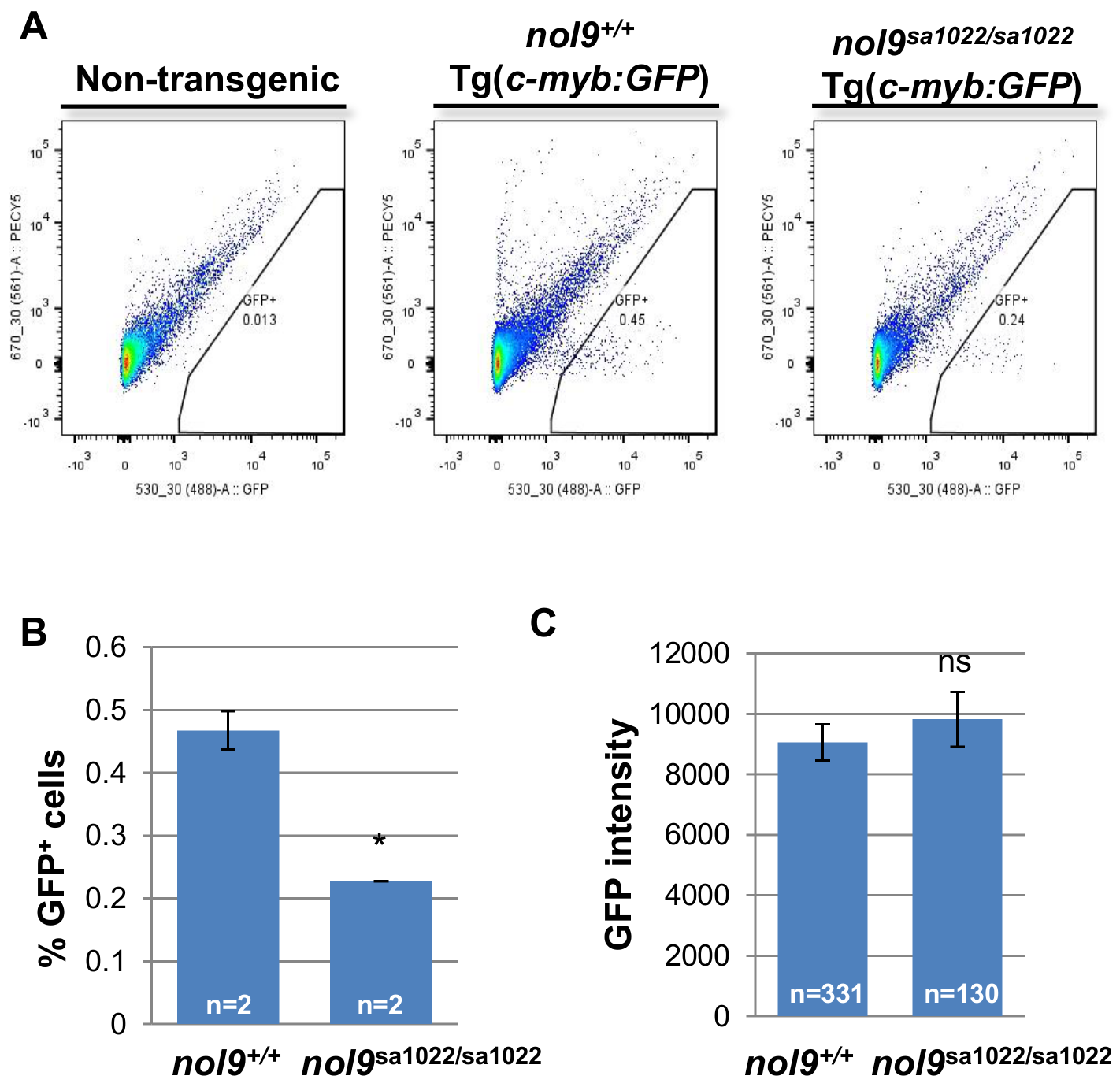

Supplement: S10 Fig — (A) Representative results of flow cytometric analysis of the number of c-myb + cells in the CHT of nol9 sa1022/sa1022 and nol9 +/+ larvae, using the progeny of a nol9 +/sa1022 Tg(c-myb:EGFP) x nol9 +/sa1022 cross at 96 hpf. Non-transgenic siblings were included as a negative control. Gating of the GFP+ population is shown. (B) The number of c-myb + cells in the CHT of 96 hpf Tg(c-myb:EGFP) nol9 sa1022/sa1022 and nol9 +/+ larvae analysed by flow cytometry. Data are represented as the percentage of GFP+ cells within the single cell population. Student’s t-Test, n = 2, *, p<0.05. (C) Average intensity of the GFP signal for c-myb + cells in the CHT of 96 hpf Tg(c-myb:EGFP) nol9 sa1022/sa1022 and nol9 +/+. Only the cells gated as shown in (A) were included. Data represented as average +/- SEM. Student’s t-Test, n = 2, p>0.05, ns–not significant. (TIFF) [file pgen.1005677.s010.tiff]

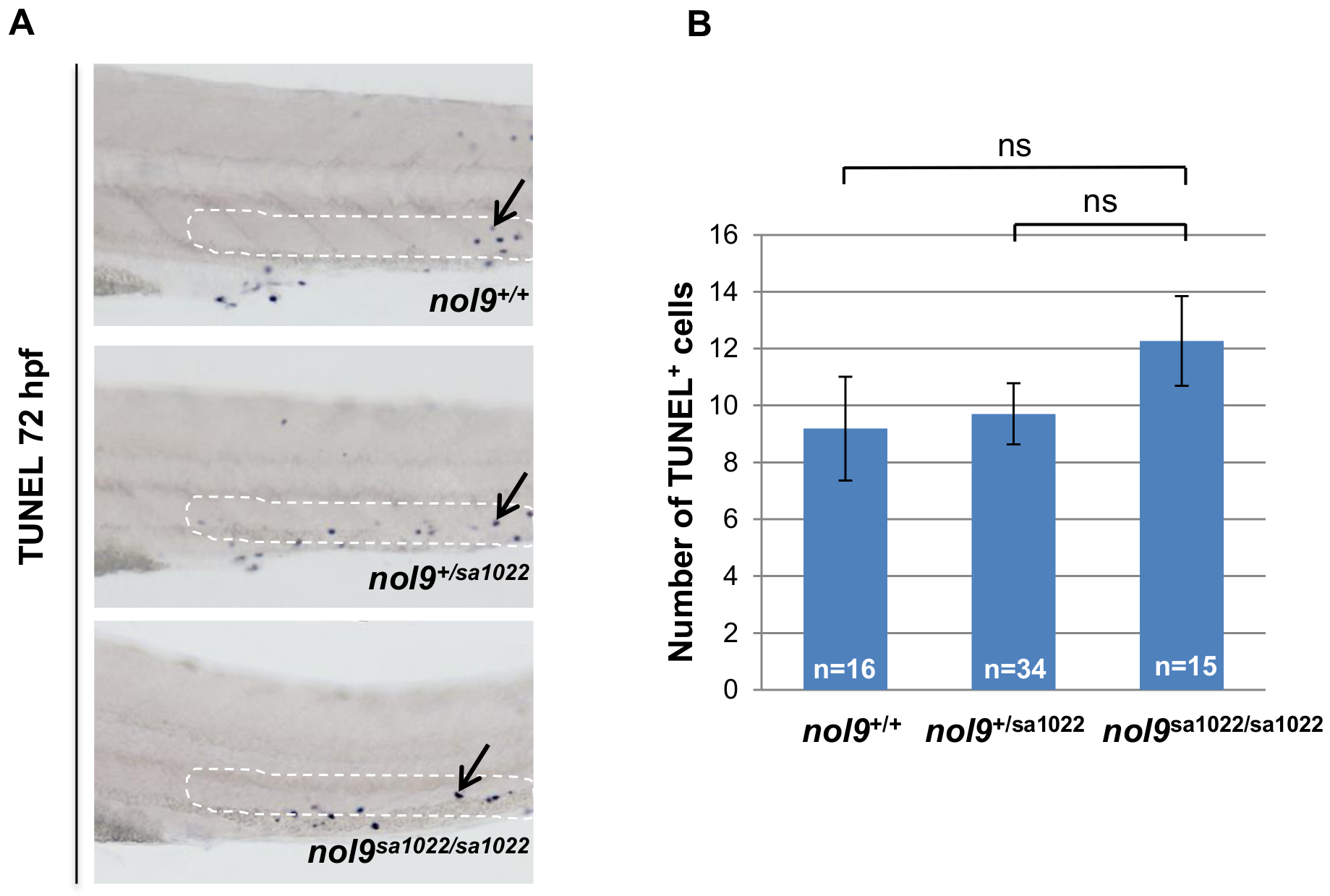

Supplement: S11 Fig — (A) The CHT region of 72 hpf larvae stained with TUNEL assay. nol9 sa1022/sa1022 mutants do not exhibit an increase in the number of TUNEL-positive apoptotic cells (arrow) compared to wt siblings in the CHT region (as outlined). All larvae are oriented with anterior to the left and dorsal to the top. (B) The average number of TUNEL-positive apoptotic cells in the CHT region in nol9 +/+ (n = 16), nol9 +/sa1022 (n = 34) and nol9 sa1022/sa1022 (n = 15) larvae at 72 hpf. Data are represented as the mean +/- SEM; One way ANOVA, ns–not significant. (TIFF) [file pgen.1005677.s011.tiff]

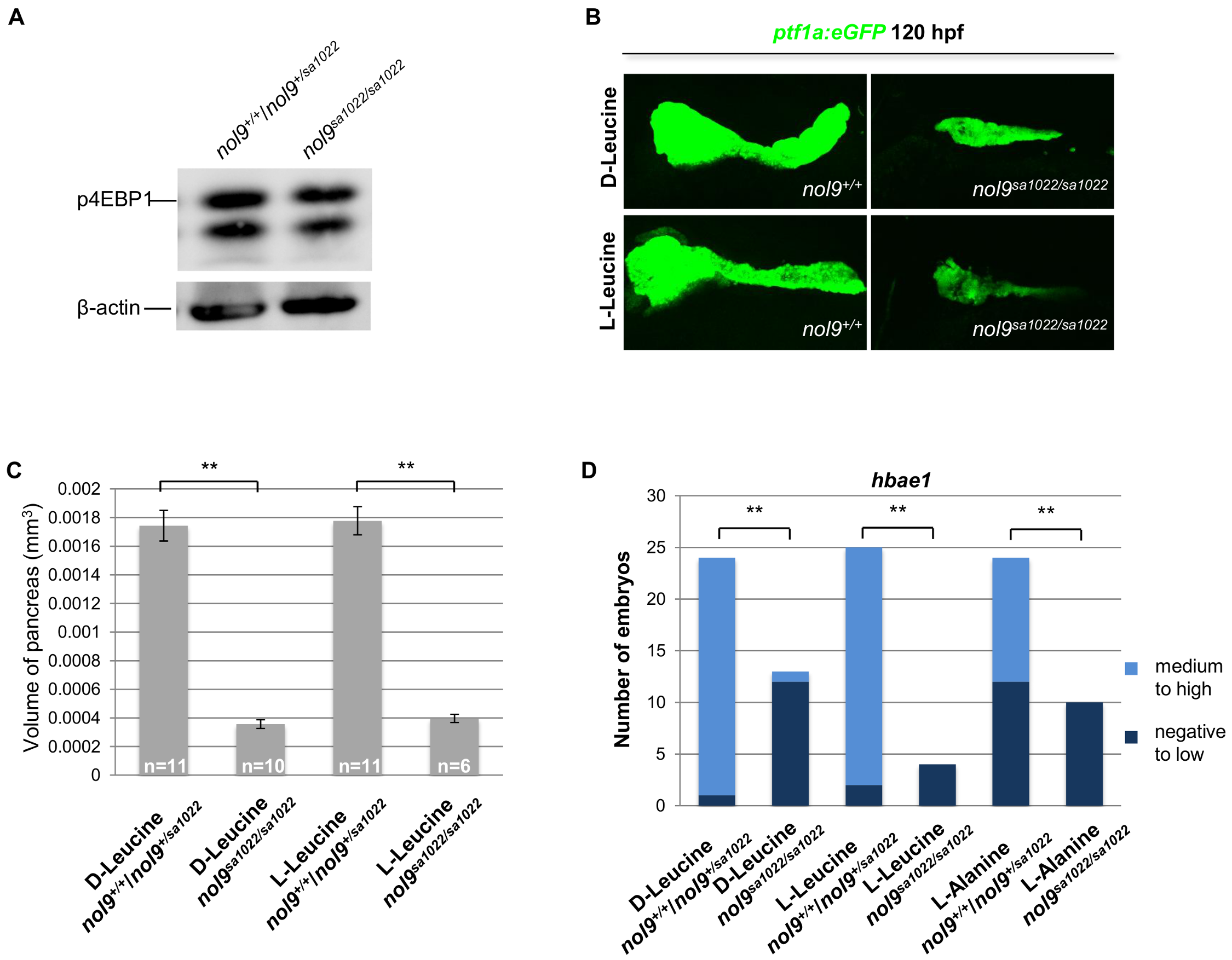

Supplement: S12 Fig — (A) Western blot analysis of p4EBP1 and β-actin (loading control) in whole cell lysates of nol9 sa1022/sa1022 and wt siblings at 120 hpf. (B) Representative confocal images of the pancreas of Tg(ptf1a:EGFP) nol9 sa1022/sa1022 and nol9 +/+ larvae at 120 hpf after treatment with L-Leucine or D-Leucine from 24 hpf. (C) The average volume of the ptf1a-positive exocrine pancreas in 120 hpf Tg(ptf1a:EGFP) larvae, depending on their genotype and treatment with either D-Leucine or L-Leucine from 24 hpf. The data are represented as the mean +/- SEM. Student’s t-test, **, p<0.01. (D) Quantification of hbae1 WISH performed on 120 hpf larvae treated with D-Leucine, L-Leucine or L-Alanine from 24 hpf. Data are represented as the number of wt (nol9 +/+/nol9 +/sa1022) or mutant (nol9 sa1022/sa1022) larvae belonging to either phenotypic group. Fisher’s exact test, **, p<0.01. (TIFF) [file pgen.1005677.s012.tiff]

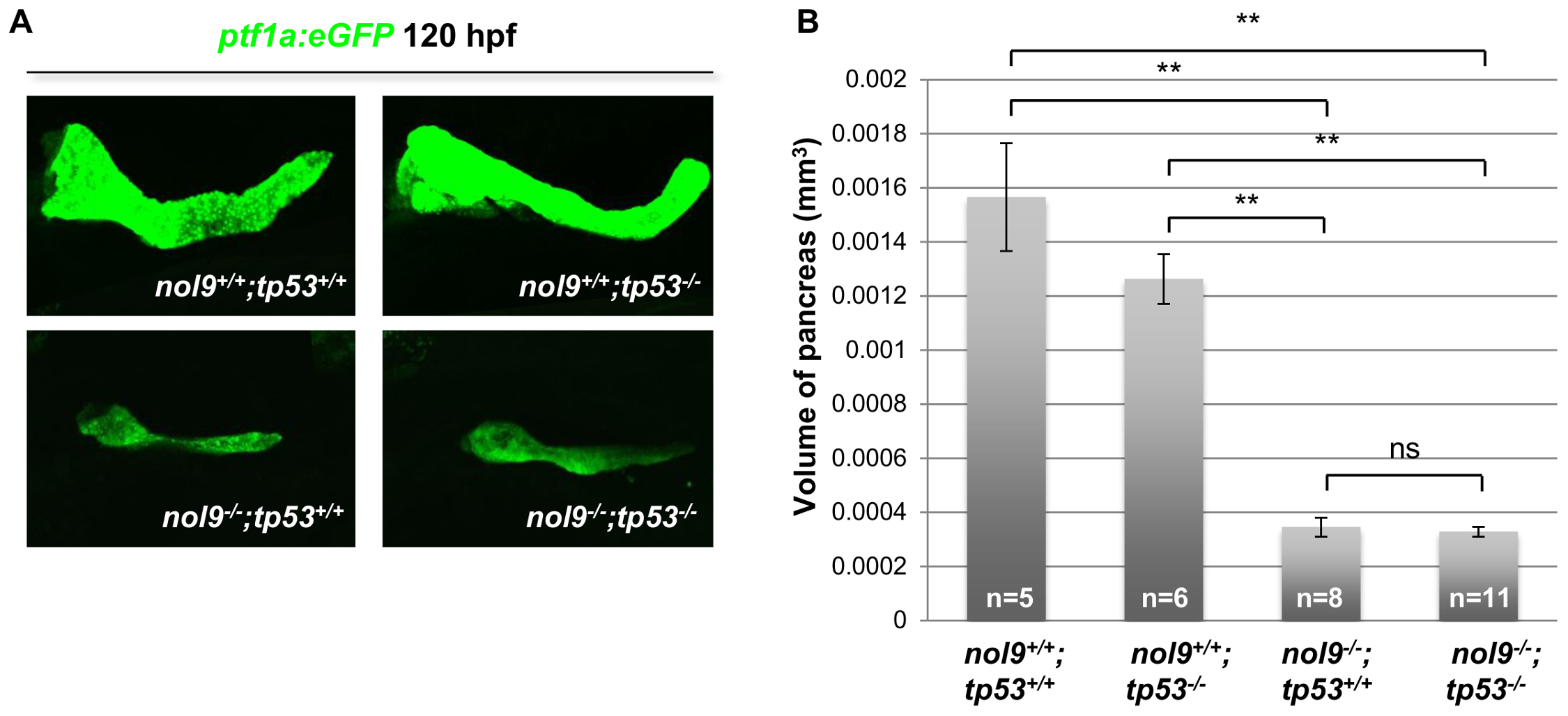

Supplement: S13 Fig — (A) Confocal images of the pancreas of 120 hpf larvae from a Tg(ptf1a:EGFP);nol9 +/sa1022 ;tp53 +/zdf1 x nol9 +/sa1022 ;tp53 +/zdf1 cross. (B) The average volume of the ptf1a + exocrine pancreas of 120 hpf larvae from a Tg(ptf1a:EGFP);nol9 +/sa1022 ;tp53 +/zdf1 x nol9 +/sa1022 ;tp53 +/zdf1 cross, depending on their genotype. Data are represented as the mean +/- SEM. Student’s t-test, **, p<0.01, ns–not significant. Within this figure, nol9 sa1022 allele has been denoted as nol9 - and tp53 zdf1 as tp53 -. (TIFF) [file pgen.1005677.s013.tiff]
